# *Lactobacillus Salivarius*‐Derived Indole‐3‐Acetic Acid Promotes AHR‐PARP1 Axis‐Mediated DNA Repair to Mitigate Intestinal Aging

**DOI:** 10.1002/advs.202515794

**Published:** 2025-10-28

**Authors:** Zheng Cao, Cui Zhang, Hehua Lei, Weichuan Lin, Wenkai Yu, Xin Gao, Yanmeng He, Xinzhi Li, Qingwei Xiang, Zhiwen Zhang, Weifei Luo, Andrew D. Patterson, Limin Zhang, Gang Chen

**Affiliations:** ^1^ State Key Laboratory of Magnetic Resonance and Imaging National Centre for Magnetic Resonance in Wuhan Innovation Academy of Precision Measurement Science and Technology Chinese Academy of Sciences (CAS) Wuhan 430071 China; ^2^ University of Chinese Academy of Sciences Beijing 100049 China; ^3^ School of Pharmacy Faculty of Medicine Laboratory for Drug Discovery from Natural Resource State Key Laboratory of Quality Research in Chinese Medicine Macau University of Science and Technology Macao 999078 China; ^4^ Hubei Shizhen Laboratory Department of Geriatrics & Department of Orthopedic Surgery Hubei Provincial Hospital of Traditional Chinese Medicine (Affiliated Hospital of Hubei University of Chinese Medicine) Wuhan 430060 China; ^5^ Guangxi Key Laboratory of Longevity Science and Technology AIage Life Science Corporation Ltd. Nanning 530200 China; ^6^ Department of Veterinary and Biomedical Sciences The Pennsylvania State University University Park PA 16802 USA; ^7^ Lead Contact

**Keywords:** aryl hydrocarbon receptor (AHR), indole‐3‐acetic acid, intestinal aging, *Lactobacillus salivarius*, poly (ADP‐ribose) polymerase 1 (PARP1)

## Abstract

Increasing evidence suggests that the aryl hydrocarbon receptor (AHR) and poly (ADP‐ribose) polymerase 1 (PARP1) are closely linked to aging and aging‐related disorders. However, the underlying mechanisms of AHR‐PARP1 axis‐mediated DNA repair in countering aging remain largely unknown. In this study, it is found that both aged humans and mice exhibit marked intestinal aging, characterized by gut dysbiosis and dysfunction and DNA damage, compared to their young counterparts. Intriguingly, it is discovered that intestinal AHR activation by indole‐3‐acetic acid (IAA), which is derived from Lactobacillus salivarius rather than host cells, effectively mitigates intestinal aging by regulating DNA‐damage responses. Mechanistically, activated AHR by IAA interacts with PARP1, potentiating PARP1 activity and the polymerization of poly (ADP‐ribose) (PARylation) by binding to its promoter. This interaction enhances intestinal barrier function and suppresses inflammation and cell senescence. Finally, the interplay between AHR and PARP1 is confirmed by in vivo and in vitro experiments, including intestine‐specific Ahr knockout mice, Ahr and Parp1 knockdown, and Parp1 overexpression in enterocytes. These findings provide a potential intervention strategy targeting AHR‐PARP1 axis to mitigate age‐related intestinal dysfunction.

## Graphical abstract

Indole‐3‐acetic acid (IAA) acting as one of the aryl hydrocarbon receptor (AHR) endogenous ligands is identified to be derived from Lactobacillus salivarius via its catalytic enzyme (ALDH) rather than host cells. Lactobacillus salivarius and IAA supplementation effectively mitigate intestinal aging by facilitating AHR‐poly (ADP‐ribose) polymerase 1 (PARP1) axis‐mediated DNA repair process.

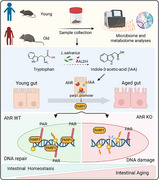

## 1 Introduction

The human gut serves as a metabolic “super organ” that is populated by trillions of microorganisms and plays critical roles in maintaining host health via gut‐host interactions.^[^ ^1^, ^2^, ^3^ ^]^ The gut microbiota is a complex and dynamic ecosystem that influences a wide range of physiological processes, including metabolic homeostasis and immune regulation.^[^ ^4^, ^5^, ^6^ ^]^ Numerous studies indicated that distinct shifts occur in the diversity, composition, and functionality of human gut microbiota during aging, a phenomenon referred to as “microbiota aging,” thereby leading to dysbiosis and susceptibility to aging‐related disorders.^[^ ^7^, ^8^, ^9^ ^]^ Notably, integrity of the intestinal barrier is important for gut health due to the preservation of intestinal epithelium cells (IECs).^[^ ^10^, ^11^ ^]^ Intestinal barrier dysfunction often results in increased intestinal permeability and gut leakiness that ultimately triggers systemic chronic inflammation and a decline in immune system function called “inflammaging” and “immune aging,” respectively.^[^ ^12^, ^13^ ^]^ Hence amelioration of intestinal aging could be critical for many aging‐related degenerative diseases such as Alzheimer's diseases, osteoporosis and sarcopenia owing to crosstalk between the gut and other host organs.^[^ ^14^, ^15^, ^16^, ^17^, ^18^, ^19^ ^]^

It is well‐documented that the aryl hydrocarbon receptor (AHR), a ligand‐activated transcription factor, is essential for maintenance of intestinal homeostasis in regulating immune response, proliferation, and differentiation of IECs.^[^ ^20^, ^21^, ^22^ ^]^ AHR activation by endogenous ligands rather than exogenous xenobiotic ligands (e.g., dioxin‐like compounds) has been shown to be beneficial for intestinal homeostasis and host health.^[^ ^23^, ^24^, ^25^, ^26^ ^]^ In the gastrointestinal tract, gut bacteria species alone or cooperation with each other can metabolize tryptophan (Trp), an essential amino acid from dietary sources, to generate indole metabolites such as indole‐3‐aldehyde (IAld), indole‐3‐ acetic acid (IAA), indole‐3‐propionic acid (IPA), indoleacrylic acid (IA), indole ethanol (IE), indole‐3‐acetaldehyde (IAAld), and indolelactic acid (ILA), most of which have been identified as endogenous AHR ligands through different catalytic enzymes.^[^ ^27^, ^28^ ^]^ For example, *Peptostreptococcus spp*. can metabolize Trp to IA and IPA.^[^ ^29^ ^]^ *Lactobacillus* (*L*.) *reuteri*, *L. johnsonii*, *L. acidophilus*, and *L. murinus* can generate IAld from Trp through the aromatic amino acid aminotransferase (ArAT) and indole lactic acid dehydrogenase (ILDH).^[^ ^30^ ^]^ Subsequently, IAld can be further converted into IAA by aldehyde dehydrogenase (ALDH)‐encoding bacterial species such as *Bifidobacterium spp*. and *Clostridium bartlettii*.^[^ ^23^, ^31^ ^]^ Conversely, AHR deficiency or disruption of AHR signaling induced significant premature senility accompanied with intestinal dysbiosis, inflammation, and impaired spatial memory in mice during aging.^[^ ^32^, ^33^, ^34^ ^]^ Previous studies revealed that inadequate AHR activation due to the decreased ability of the microbiota to produce AHR ligands highly contributes to many age‐related disorders such as high blood pressure, diabetes, and neurodegenerative diseases.^[^ ^35^, ^36^ ^]^ Therefore, targeted strategies to regulate AHR activity and Trp metabolite production will be beneficial for amelioration of aging and age‐related diseases.^[^ ^37^, ^38^, ^39^ ^]^ However, which specific bacterial strain possess a catalytic capacity to produce corresponding Trp metabolites remain to be identified and their physiological functions during aging need to be full explored.

The aging process involves DNA damage, protein misfolding, and organelle degeneration.^[^ ^40^, ^41^, ^42^ ^]^ The key ratio is between accumulation of damage and the compensation mechanism to repair the damage.^[^ ^43^ ^]^ DNA integrity is essential for maintaining cellular function and organismal health, yet it becomes progressively compromised with age due to cumulative damage and declining repair capacity.^[^ ^44^ ^]^ Accumulation of DNA damage including single‐strand breaks (SSBs) and double‐strand breaks (DSBs) is a hallmark of aging that contributes to genomic instability underlying many age‐related pathologies, such as cancer and neurodegeneration.^[^ ^45^, ^46^ ^]^ Poly(ADP‐ribose) polymerase 1 (PARP1), a central sensor and mediator of DNA repair, plays a pivotal role in preserving genomic integrity.^[^ ^47^ ^]^ Upon sensing DNA damage, PARP1 is rapidly activated and orchestrates DNA repair by recruiting downstream effectors involved in base excision repair (BER) and single‐strand break repair (SSBR).^[^ ^48^, ^49^ ^]^ However, chronic activation of PARP1 in response to continuous DNA damage occurring in aging cells induces excessive NAD⁺ consumption, thus driving cellular energy imbalance, metabolic dysfunction and mitochondrial impairment.^[^ ^50^ ^]^ Aberrant PARP1 signaling has also been implicated in inflammaging, and its chronic activation promotes the release of pro‐inflammatory mediators via the NF‐κB pathway.^[^ ^51^, ^52^ ^]^ Therefore, PARP1 exhibits dual roles in safeguarding genomic stability and contributing to metabolic dysfunction that highlight its complex involvement in aging.^[^ ^53^, ^54^ ^]^ In view of the close connection between intestinal AHR signaling, DNA damage and aging, we hypothesized that microbiota‐derived Trp metabolites may regulate intestinal aging by facilitating AHR‐PARP1 signaling axis‐mediated DNA repair process.

In this study, our clinical cohort and animal experiments showed that relatively old humans and mice exhibited lower abundance of *Lactobacillus salivarius* (*L. salivarius*) and IAA than their respective young counterparts. In vivo and in vitro experiments identified that *L. salivarius* produces IAA via its catalytic enzyme (ALDH) instead of host cells. Further, we investigated the mechanisms by which *L. salivarius*‐derived IAA alleviates intestinal aging by examining intestinal barrier functions and DNA repair in mice. Collectively, these findings reveal that AHR activation by IAA can effectively alleviate intestinal aging by driving PARP1‐mediated DNA repair process.

## 2 Results

### 2.1 Alteration of Gut Bacteria and Trp Metabolism with Human and Mouse Aging

**Figure 1:**
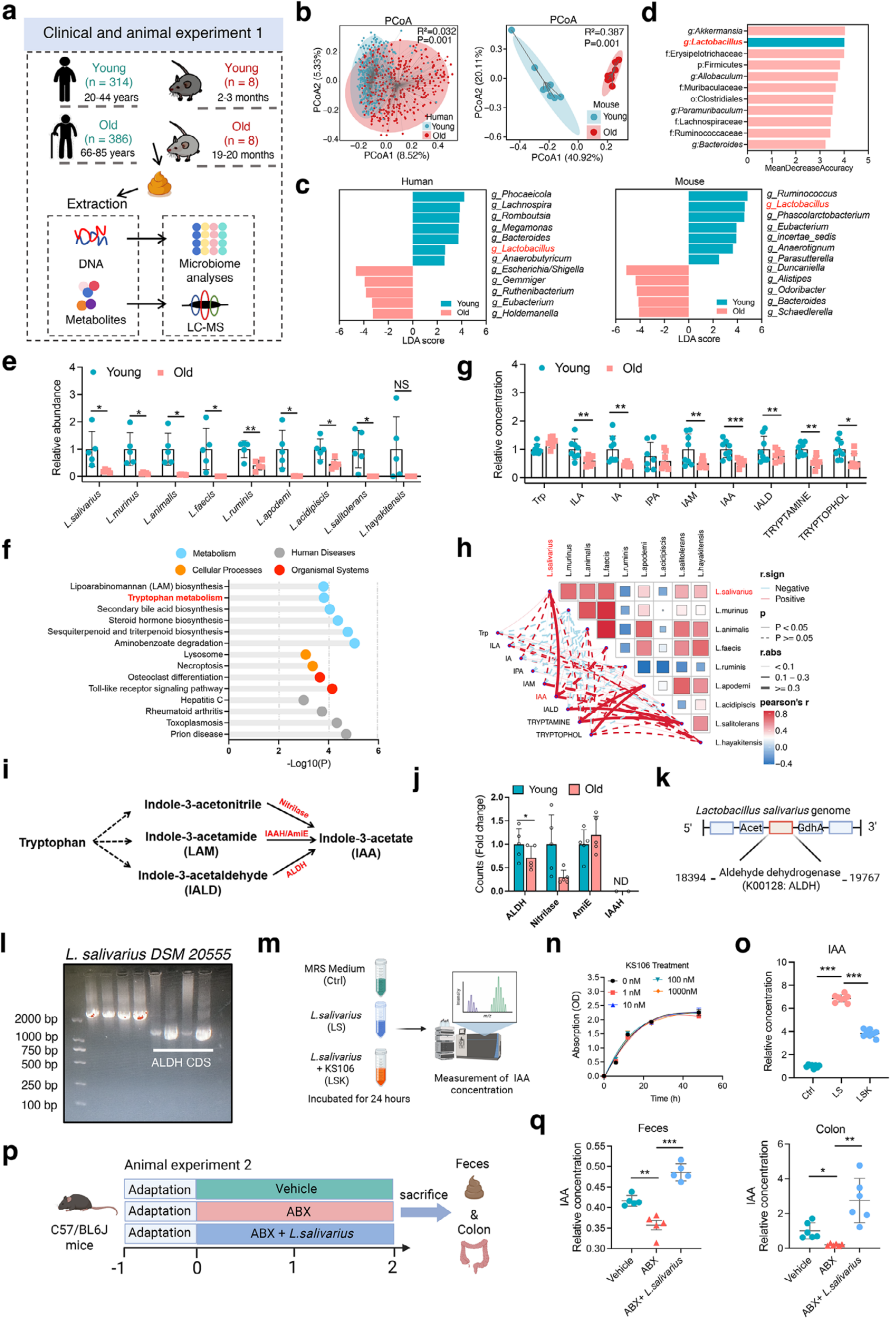
Identification of *L. salivarius* and its metabolite IAA serving as microbial and metabolic biomarkers in aging. a) Schematic overview of clinical and animal experiment 1. Fecal samples were collected from young (20–44 years, *n* = 314) and old (66–85 years, *n* = 386) human subjects, as well as young (2–3 months, *n* = 8) and old (19–20 months, *n* = 8) mice for 16S rRNA gene sequencing and metabolomic analysis. b) 𝛽‐diversity index analysis based on Bray–Curtis dissimilarities (PCoA). c) Linear discriminant analysis (LDA) identifying differential genera. d) Random forest analysis highlighting key genera associated with age in mouse samples. e) Relative abundances of *Lactobacillus* species in young and old feces (mean ± SD, *n* = 5 per group). f) Differential pathways identified by metagenomic functional annotation, showing altered tryptophan metabolism and related pathways between young and old groups. g) Quantification of fecal tryptophan metabolites by LC‐MS in young and old groups (mean ± SD, *n* = 8). h) Correlation heatmap showing associations between *Lactobacillus* species abundance and fecal tryptophan metabolites. i) Schematic of tryptophan metabolic pathways inducing indole‐3‐acetate (IAA) production via nitrilase, acetaldehyde dehydrogenase (ALDH) and IAAH/Amidase enzymes catalysis. j) Relative gene counts related to tryptophan metabolism (ALDH, nitrilase, Amide hydrolase, and IAAH) in young and old groups. ND, not detected (mean ± SD, *n* = 5 per group). k) Schematic of the identified ALDH gene locus in the *Lactobacillus salivarius* genome. l) PCR amplification of ALDH encoding sequence from *L. salivarius DSM 20* *555*. m) Experimental setup for measuring IAA production in MRS medium (Ctrl), *L. salivarius* culture (LS), and *L. salivarius* treated with ALDH inhibitor KS106 (LSK). n) Growth curve of *L. salivarius* treated with KS106 at different dosages measured by optical density (OD) at 600 nm (*n* = 3 per group). o) Relative IAA concentration measured in Ctrl, LS, and LSK groups (*n* = 3 per group). p) Schematic of animal experiment 2: aged mice were treated with antibiotics (ABX) or ABX followed by *L. salivarius* gavage for two weeks. q) Quantification of IAA among PBS, ABX, and ABX + *L. salivarius* groups in fecal and colon (mean ± SD, *n* = 6 per group). Statistical significance was determined by unpaired two‐tailed Student's t‐test or one‐way ANOVA with Tukey's post hoc test. *P < 0.05, **P < 0.01, ***P < 0.001.

To investigate the alteration of gut microbial community and microbiota‐related metabolism, we collected fecal samples from young (20–44 years old, *n* = 314) and old (66–85 years old, *n* = 386) human subjects and cecal contents from young (2–3 months, *n* = 8) and old (19–20 months, *n* = 8) mice (**Figure** **1**a). 16S rRNA gene sequencing analysis revealed that both aged human and mice exhibited profound changes in the gut microbiota community, characterized by significantly altered microbial α‐diversity with increased ACE index and decreased Shannon's diversity index (Figure S1a, Supporting Information). The principal coordinate analysis (PCoA) of microbial β‐diversity based on Bray‐Curtis distances indicated that both human (R^2^ = 0.032, P = 0.001) and mice (R^2^ = 0.387, P = 0.001) with aging also showed distinct shifts in total population of the gut microbiota (Figure 1b), which was further confirmed by microbial composition changes at the phylum level (Figure S1b, Supporting Information). Of particular note, the relative abundance of family *Lactobacillaceae* and genus *Lactobacillus* were strikingly downregulated in aged humans and mice compared with their young counterparts, as revealed by linear discriminant analysis (LDA) (Figure 1c), random forest analysis (Figure 1d), and LEfSe cladogram representing the relative abundance of each genus (Figure S1c–f, Supporting Information). Metagenomic sequencing analysis further demonstrated that aged mice exhibited significant reductions in the abundance of multiple *Lactobacillus* spp, including *L. salivarius*, *L. murinus*, *L. animalis*, *L. faecis*, *L. ruminis*, *L. apodeme*, *L. acidipiscis*, *L. salitolerans*, and *L. hayakitensis* (Figure 1e). No significant differences were observed in the relative abundances of *L. plantarum* and *L. casei* being the most extensively studied species between young and aged mice. Interestingly, metagenomic functional annotation revealed that pathways such as bile acid metabolism, steroid, and steroid hormone biosynthesis were markedly altered in aged mice in comparison with young mice, especially downregulation of Trp metabolism (Figure 1f). HPLC‐QQQ‐MS‐based targeted metabolomic quantification indicated that aged mice exhibited significantly lower levels of indole metabolites such as ILA, IA, IAM, IAA, and IAld than young controls (Figure 1g). Pearson correlation analysis showed a comprehensive association between the levels of most indole metabolites and genus of gut bacteria to varying degrees in human and mice samples (Figure S3, Supporting Information). Notably, the abundance of multiple *Lactobacillus* species such as *L. salivarius* exhibited significantly positive correlations with the level of IAA (Figure 1h). Together, these results suggest that *L. salivarius* and IAA are closely associated with the improved intestinal homeostasis of mice during aging.

### 2.2 *L. salivarius* Instead of Host Cells Produces IAA via Bacterial ALDH

Given a positive correlation between *L. salivarius* and IAA, we next investigated whether or how *L. salivarius* is responsible for IAA production. Normally, Trp metabolism via the gut microbiota produces IAA from the precursor compounds within several metabolic pathways that are catalyzed by respective enzymes including nitrilase, aldehyde dehydrogenase (ALDH), indole‐3‐acetaldehyde hydrolase (IAAH) and amide hydrolase (AmiE) (Figure 1i). Gene abundance analysis of metagenomic sequencing revealed that gene counts of ALDH were significantly reduced in aged mice, whereas nitrilase, AmiE, and IAAH showed no significant differences in the gene counts between young and old groups (Figure 1j). Further genomic analysis identified the presence of ALDH (K00128)‐encoding gene sequences from 18 394 bp to 19 767 bp within the *L. salivarius* genome (Figure 1k) that was confirmed by PCR amplification of ALDH encoding sequence in *L. salivarius* DSM 20 555 strain (Figure 1l). However, other *Lactobacillus* species within *Lactobacillus* genus lack K00128‐encoding ALDH gene.

To assess the functional role of bacterial ALDH in IAA production, we performed *L. salivarius* culture and IAA concentration measurement under the conditions with and without treatment of ALDH inhibitor KS106 (Figure 1m). The results of growth curve analysis demonstrated that KS106 treatment did not significantly affect *L. salivarius* proliferation (Figure 1n). Compared with control, IAA production was markedly increased in *L. salivarius* culture and reduced upon ALDH inhibition by KS106 (Figure 1o). In vivo experiments further showed that daily oral gavage with *L. salivarius* for 7 days led to significant elevations in the levels of fecal *L. salivarius* and IAA (Figure S4a–c, Supporting Information). Meanwhile, *L. salivarius* supplementation to aged mice with antibiotics (ABX) treatment markedly restored the levels of Trp metabolites, especially IAA and IPA, which were significantly depleted in ABX‐treated controls (Figure 1p,q; Figure S2, Supporting Information). To verify whether host IECs can catalyze the conversion of Trp intermediates to IAA, Caco‐2 cells were incubated with IAld, a direct substrate for ALDH‐mediated IAA synthesis (Figure s4 d, Supporting Information). No significant changes in IAA levels were observed after incubation of IALD for 24 h (Figure S4e, Supporting Information). Similarly, no signals of ^13^C‐labeled IAA were detected in Caco‐2 cells incubated with isotopically labeled ^13^C‐Trp (Figure S4f,g, Supporting Information). In addition, BLAST analysis showed that there is only 33% similarity of ALDH gene sequence between *L. salivarius* and human (Figure S5, Supporting Information). Collectively, these in vitro and in vivo results indicate that *L. salivarius* residing in the gut produces IAA via its own ALDH rather than host intestinal epithelial ALDH.

### 2.3 Colonization of Mice with Live *L. salivarius* Effectively Mitigates Intestinal Aging

**Figure 2:**
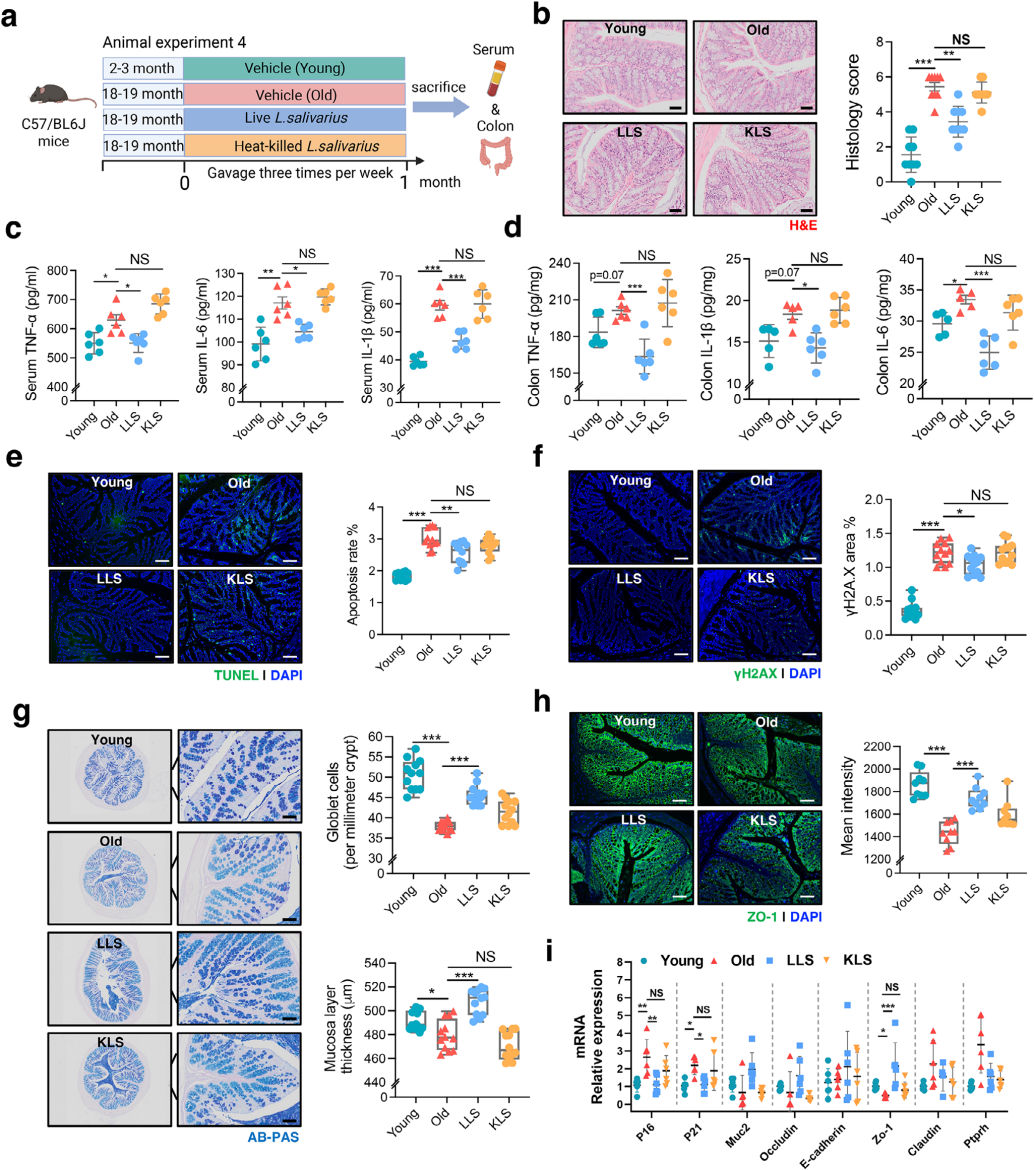
*L. salivarius* supplementation ameliorates intestinal aging phenotype. a) Schematic of animal experiment 4: Young (2–3 months) and aged (18–19 months) mice were treated with vehicle, live *L. salivarius* (LLS), or heat‐killed *L. salivarius* (KLS) for one month, followed by collection of colon and serum samples (*n* = 6 per group). b) Representative images of H&E staining of colon tissues. (Scale bar = 50 µm, *n* = 3 per group). c) Serum levels of TNF‐α, IL‐6, and IL‐1β measured by ELISA in each group (*n* = 6 per group). d) Colon tissue levels of TNF‐α, IL‐6, and IL‐1β measured by ELISA. e) Representative TUNEL staining images of colon sections showing apoptotic cells (green) and nuclei (DAPI, blue) (Scale bar = 50 µm), and quantification of apoptotic cell percentage (right) (*n* = 3 mice per group with three random fields per section). f) Representative γH2AX immunofluorescence staining of colon sections showing DNA damage (green) and nuclei (DAPI, blue) (Scale bar = 50 µm), and quantification of γH2AX‐positive area (%) in colon tissues (right) (*n* = 3 mice per group with three random fields per section). g) Representative images of AB‐PAS staining at 4× and 20× magnification showing goblet cells and mucus layer structure in colonic tissues (Scale bar = 50 µm), and quantification of goblet cell numbers (per millimeter of crypt length) in the colon across groups (right) (*n* = 3 mice per group with three random fields per section) h) Representative ZO‐1 immunofluorescence staining of colon sections (Scale bar = 50 µm), and quantification (*n* = 3 mice per group with three random fields per section). i), Relative mRNA expression levels of *P16*, *P21*, *Muc2*, *Occludin*, *E‐cadherin*, *Zo‐1*, *Claudin*, and *Ptprh* genes in colon tissues measured by qRT‐PCR (mean ± SD, *n* = 6 per group). Statistical significance was determined by one‐way ANOVA followed by Tukey's post hoc test. *P < 0.05; **P < 0.01; ***P < 0.001; NS, no significance.

To evaluate whether *L. salivarius* colonization can ameliorate intestinal aging, aged mice (18–19 months) were orally administered with live or heat‐killed *L. salivarius* (or vehicle control) twice per week for one month (**Figure** **2**a). Histological H&E (Figure 2b) and AB‐PAS staining analyses (Figure 2g) of intestinal tissues showed that supplementation with live *L. salivarius* (LLS), but not heat‐killed bacteria (KLS), partially restored intestinal structure, manifested by significant upregulation of the number of goblet cells per crypt and mucosal layer thickness that were markedly diminished in aged mice (Figure 2g). Concurrently, supplementation of LLS instead of KLS significantly reduced the levels of pro‐inflammatory cytokines (TNF‐α, IL‐6, and IL‐1β) in both serum and colon tissues (Figure 2c,d), which were triggered by high level of LPS due to the barrier dysfunction and bacterial translocation of age mice (Figure S6a, Supporting Information). Immunofluorescence staining with TUNEL and γ‐H2A histone family member X (γH2AX), a known marker related to DNA damage and repair, demonstrated that supplementation of LLS rather than KLS markedly attenuated cell apoptosis and DNA damage in intestinal epithelium of aged mice (Figure 2e, f). Moreover, RT‐qPCR analysis revealed that LLS supplementation significantly downregulated mRNA levels of typical cell senescence markers (*p16* and *p21*) and key intestinal barrier–associated genes (*Muc2* and *Zo‐1*) (Figure 2h, i). In addition, only LLS supplementation restored the concentration of IAA in both colon and feces of aged mice (Figure S8a,b, Supporting Information). In summary, these findings indicate that live *L. salivarius* can effectively alleviate intestinal aging phenotype probably participating in repairment of gut barrier function.

### 2.4 *L. salivarius*‐Derived IAA Mitigates Intestinal Aging Phenotypes Dependent on AHR Signaling In Vivo and In Vitro

**Figure 3:**
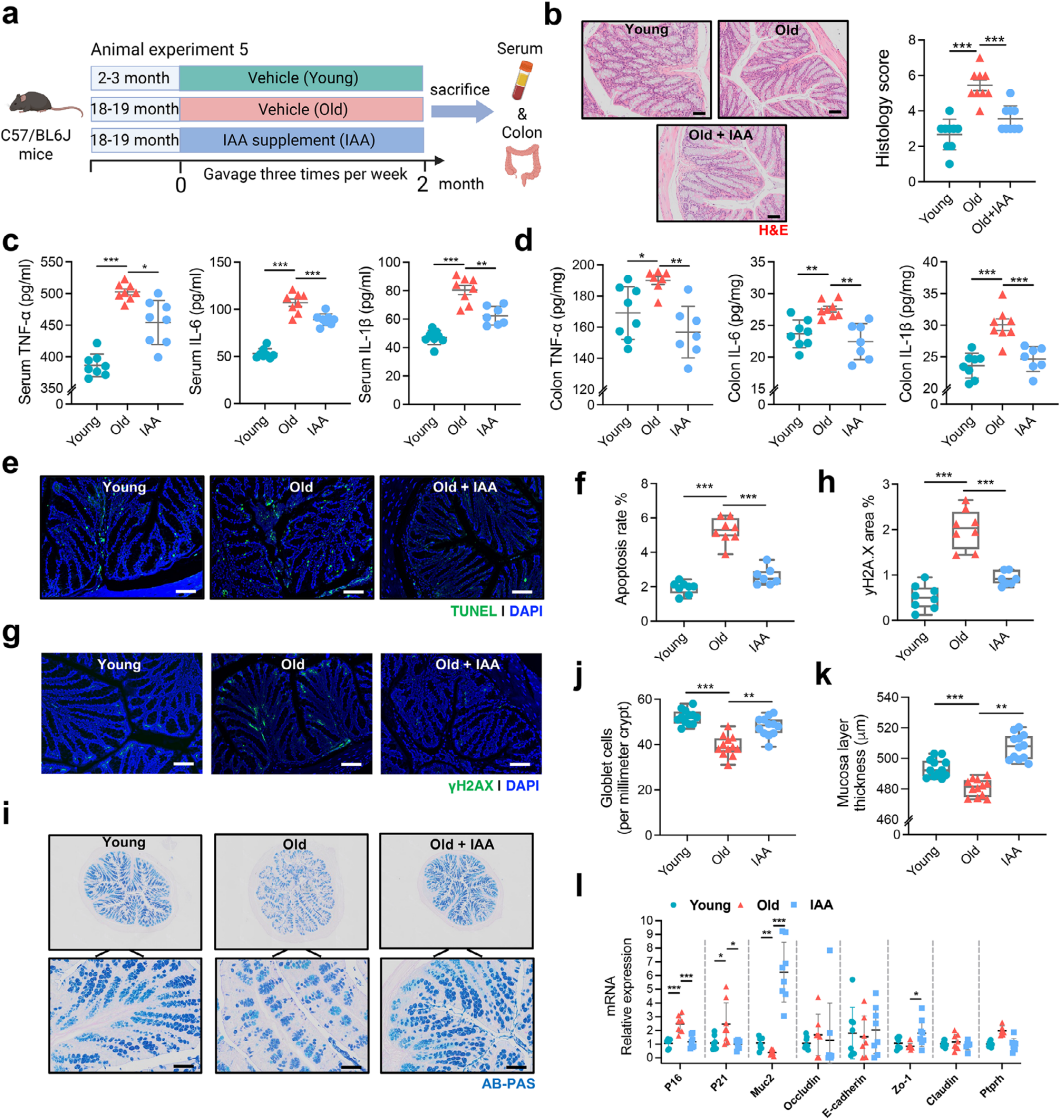
*L. salivarius*‐derived IAA supplementation mitigates intestinal aging phenotype. a) Schematic of animal experiment 5. Young (2–3 months) and aged (18–19 months) mice were treated with vehicle or IAA supplementation (50 mg kg^−1^) twice per week for two months before sacrifice and collection of colon and serum samples (*n* = 8 per group). b) Representative images of H&E staining of colon tissues from young, old, and IAA‐treated mice (Scale bar = 50 µm, *n* = 3 per group). c) Serum levels of TNF‐α, IL‐6, and IL‐1β measured by ELISA across groups (*n* = 6 per group). d) Colon tissue levels of TNF‐α, IL‐6, and IL‐1β measured by ELISA across groups (*n* = 6 per group). e) Representative TUNEL staining images of colon tissues, showing apoptotic cells (green) and nuclei (DAPI, blue) (Scale bar = 50 µm, *n* = 3 per group). f) Quantification of TUNEL‐positive apoptotic area (%) in colon sections among groups (*n* = 3 mice per group with three random fields per section). g) Representative γH2AX immunofluorescence staining images of colon tissues indicating DNA damage (green) and nuclei (DAPI, blue) (Scale bar = 50 µm, *n* = 3 per group). h) Quantification of γH2AX‐positive area (%) in colon sections among groups (*n* = 3 mice per group with three random fields per section). i) Representative images of AB‐PAS staining at 4× and 20× magnification showing goblet cells and mucus layer morphology in colon sections (Scale bar = 50 µm, *n* = 3 per group). j,k) Quantification of goblet cell numbers (per millimeter of crypt length) and mucus layer thickness among groups (*n* = 3 mice per group with three random fields per section). l) Relative mRNA expression levels of *P16*, *P21*, *Muc2*, *Occludin*, *E‐cadherin*, *Zo‐1*, *Claudin*, and *Ptprh* in colon tissues measured by qRT‐PCR. (mean ± SD, *n* = 8 mice per group). Statistical significance was assessed by one‐way ANOVA followed by Tukey's post hoc test. *P < 0.05; **P < 0.01; ***P < 0.001; NS, no significance.

**Figure 4:**
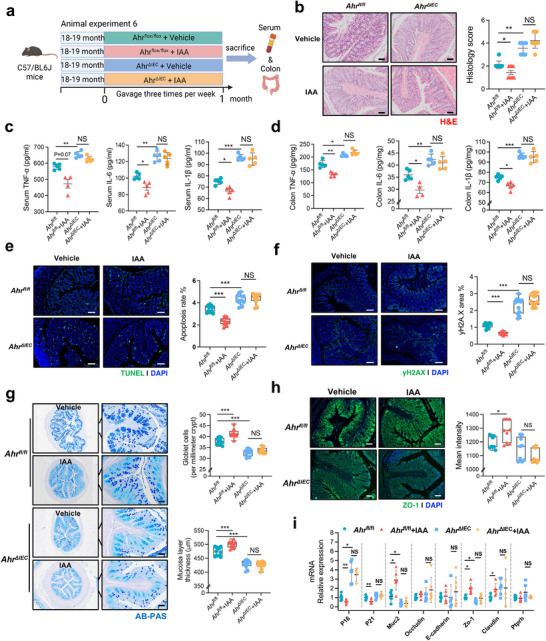
IAA supplementation mitigates intestinal aging dependent on intestinal AHR signaling in vivo. a) Schematic of animal experiment 6: aged (18–19 months) *Ahr* ^fl/fl^ and *Ahr* ^ΔIEC^ mice were treated with vehicle or IAA supplementation (50 mg kg^−1^) twice per week for two months prior to colon and serum sample collection (*n* = 6 per group). b) Representative H&E staining images of colon tissues from different groups (20 × magnification, *n* = 3 per group). c) ELISA measurements of serum TNF‐α, IL‐6, and IL‐1β levels (n = 6 per group). d) ELISA measurements of colonic TNF‐α, IL‐6, and IL‐1β levels (*n* = 6 per group). e) Representative TUNEL staining images of colon sections showing apoptotic cells (green) and nuclei (DAPI, blue) (Scale bar = 50 µm), and quantification of apoptotic cell percentage (right) (*n* = 3 mice per group with three random fields per section). f) Representative γH2AX immunofluorescence staining of colon sections showing DNA damage (green) and nuclei (DAPI, blue) (Scale bar = 50 µm), and quantification of γH2AX‐positive area (%) in colon tissues (right) (*n* = 3 mice per group with three random fields per section). g) Representative images of AB‐PAS staining at 4× and 20× magnification showing goblet cells and mucus layer structure in colonic tissues (Scale bar = 50 µm), and quantification of goblet cell numbers (per millimeter of crypt length) in the colon across groups (right) (*n* = 3 mice per group with three random fields per section) h) Representative ZO‐1 immunofluorescence staining of colon sections (Scale bar = 50 µm), and quantification (*n* = 3 mice per group with three random fields per section). i) Relative mRNA expression levels of *P16*, *P21*, *Muc2*, *Occludin*, *E‐cadherin*, *Zo‐1*, *Claudin*, and *Ptprh* genes in colon tissues measured by qRT‐PCR (mean ± SD, *n* = 6 per group). Statistical significance was determined by one‐way ANOVA followed by Tukey's post hoc test. *P < 0.05; **P < 0.01; ***P < 0.001; NS, no significance.

Given that *L. salivarius* producing IAA can improve intestinal aging, we next examined whether and how IAA can ameliorate aging‐associated intestinal decline. Pharmacokinetic profiling of IAA showed that intestinal levels of IAA were much higher than that in liver of mice upon oral exposure at different dosages (20 and 200 mg kg^−1^) (Figure S7, Supporting Information). Following IAA supplementation (50 mg kg^−1^) three times per week for two months (**Figure** **3**a), aged mice showed significantly improved intestinal histological structure and related pathophysiological indicators. Intestinal H&E (Figure 3b) and AB‐PAS staining (Figure 3i) indicated that IAA supplementation restored mucosal barrier integrity, shown with increased goblet cell numbers and mucus layer thickness, which were both diminished in aged mice (Figure 3j,k). Meanwhile, high levels of pro‐inflammatory cytokines (TNF‐α, IL‐6, and IL‐1β) in serum and colon tissues of aged mice were markedly reduced (Figure 3c,d) partly due to the significantly lowered serum LPS levels (Figure S6b, Supporting Information) upon IAA supplementation. Furthermore, TUNEL and γH2AX immunofluorescence staining demonstrated that IAA treatment significantly reduced epithelial apoptosis and DNA damage of aged mice (Figure 3e–h). IAA supplementation significantly downregulated mRNA levels of senescence markers (*p16* and *p21*) and restored the expression of several key intestinal barrier–associated genes, such as *Muc2* and *Zo‐1* (Figure 3l). However, IAA supplementation did not exhibit significant improvement in intestinal dysfunction of aged mice with intestine‐specific knockout of AHR (*Ahr* ^ΔIEC^) (**Figure** **4**a), manifested by no marked restoration in intestinal morphology (Figure 4b), pro‐inflammatory cytokines (TNF‐α, IL‐6, and IL‐1β) (Figure 4c,d), goblet cell numbers and mucus layer thickness (Figure 4g), LPS levels in serum and intestine tissue (Figure S6c, Supporting Information). Importantly, aged *Ahr* ^ΔIEC^ mice upon IAA supplementation showed similar intestinal epithelial apoptosis and DNA damage to aged *Ahr* ^ΔIEC^ mice, indicated by no significant changes in TUNEL and γH2AX immunofluorescence staining (Figure 4e,f), related senescence markers (*p16* and *p21*) and intestinal barrier function–related genes (*Muc2*, *Zo‐1*, and *Claudin*) (Figure 4h,i).

**Figure 5:**
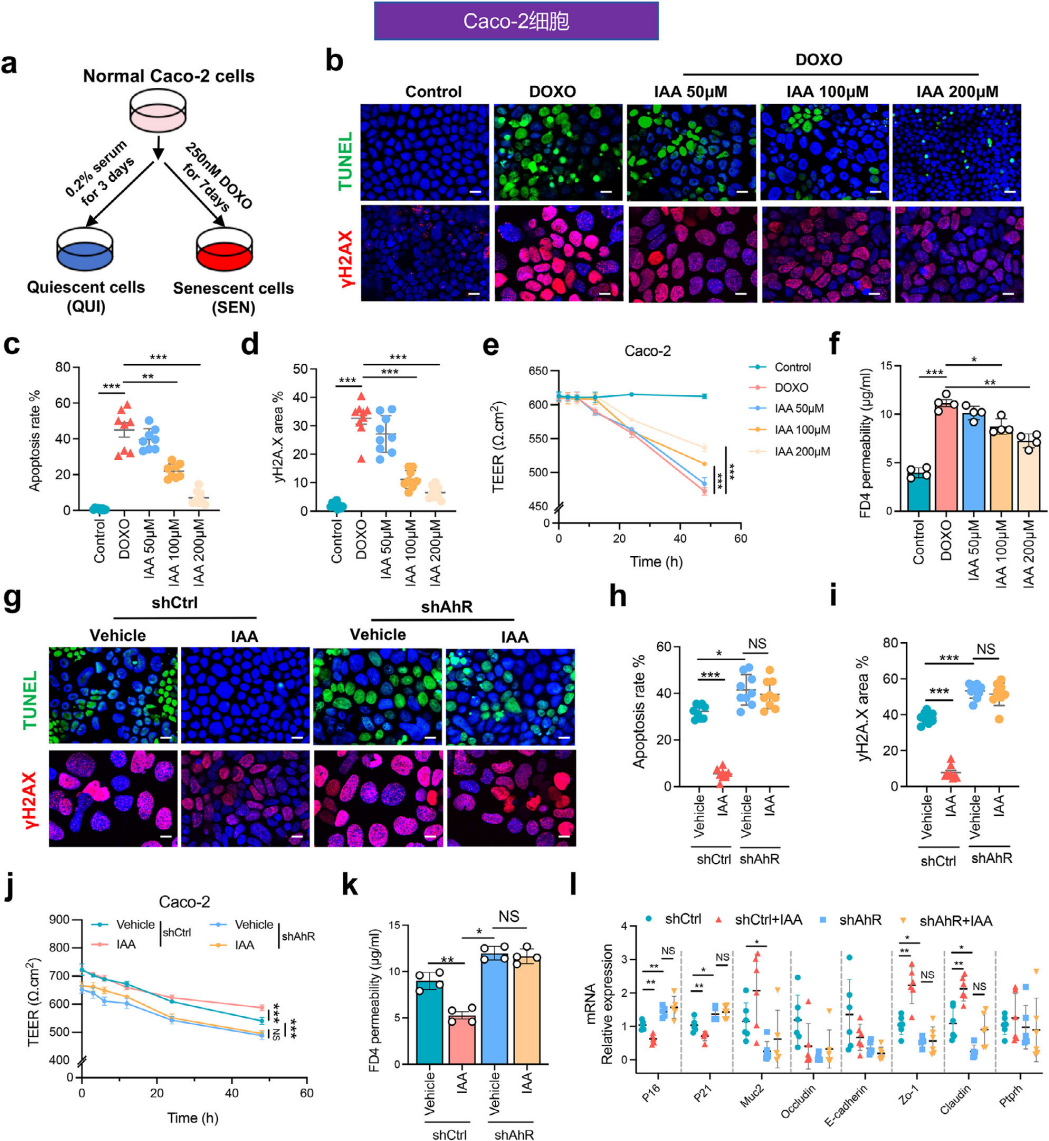
IAA supplementation mitigates intestinal aging and promotes DNA repair in AHR dependent manner in vitro. a) Schematic of cellular modeling: quiescent (QUI) and senescent (SEN) Caco‐2 cell models were established by serum starvation (0.2% FBS for 3 days) or doxorubicin (DOXO, 250 nm for 7 days) treatment, respectively. b) Representative TUNEL (green) and γH2AX (red) immunofluorescence staining of Caco‐2 cells (Scale bar = 25 µm, *n* = 3 per group). c) Quantification of apoptosis rate (%) in Caco‐2 cells (n = 3 slides per group with three random fields per section). d) Quantification of γH2AX‐positive area (%) in Caco‐2 cells (*n* = 3 slides per group with three random fields per section). e) Time‐course measurement of transepithelial electrical resistance (TEER) in Caco‐2 cells (*n* = 4 per group). f) Quantification of FD‐4 permeability in Caco‐2 cells (*n* = 4 per group). g) Representative TUNEL and γH2AX immunofluorescence images of control (NC) and shAHR knockdown Caco‐2 cells treated with vehicle or IAA (Scale bar = 25 µm, *n* = 3 per group). h) Quantification of apoptosis rate (%) in NC and shAHR Caco‐2 cells with and without IAA treatment (*n* = 3 slides per group with three random fields per section). i) Quantification of γH2AX‐positive area (%) in NC and shAHR Caco‐2 cells with and without IAA treatment (*n* = 3 slides per group with three random fields per section). j) Time‐course measurement of TEER in NC and shAHR Caco‐2 cells treated with vehicle or IAA (*n* = 4 per group). k) Quantification of FD‐4 permeability in NC and shAHR Caco‐2 cells treated with vehicle or IAA (*n* = 4 per group). l) Relative mRNA expression levels of DNA repair‐ and barrier‐associated genes (mean ± SD, *n* = 6 per group). Statistical significance was determined by one‐way ANOVA followed by Tukey's post hoc test. *P < 0.05; **P < 0.01; ***P < 0.001; NS, no significance.

To further validate the role of IAA in alleviating intestinal aging, we established an in vitro aging model using Caco‐2 cells treated with doxorubicin (DOXO, 250 nm) for 7 days (**Figure** **5**a). TUNEL and γH2AX immunofluorescence staining revealed that DOXO treatment markedly induced cell apoptosis and DNA damage, both of which were dose‐dependently reduced by IAA supplementation at different concentrations (50, 100, and 200 µm) (Figure 5b). Quantitative analyses showed significant decreases in apoptosis rates and γH2AX‐positive area of aged cells following IAA treatment (Figure 5c,d). Measurement of transepithelial electrical resistance (TEER) and FD4 permeability assays demonstrated that IAA supplementation significantly restored epithelial barrier integrity in DOXO‐treated Caco‐2 cells (Figure 5e,f). Notably, IAA supplementation failed to reduce cell apoptosis, DNA damage, TEER decline, or FD4 hyperpermeability in DOXO‐treated Caco‐2 cells with stable *Ahr* knockdown (shAHR) (Figure 5g–k; Figure S9a, Supporting Information). Moreover, RT‐qPCR analysis suggested that markedly transcriptional restoration of senescence markers (*p16* and *p21*) and gut barrier–associated genes (*Muc2*, *Zo‐1*, and *Claudin*) in senescent cells (SEN) upon IAA treatment was abolished in shAHR cells (Figure 5l). These in vivo and in vitro results indicate that IAA displays marked alleviation effects on aging‐related intestinal inflammation, barrier dysfunction, cell apoptosis and DNA damage of both aged mice and senescent cells, which are critically dependent on AHR signaling.

### 2.5 Activation of AHR by IAA Mitigates Intestinal Aging by Promoting PARP1‐Mediated DNA Repair

**Figure 6:**
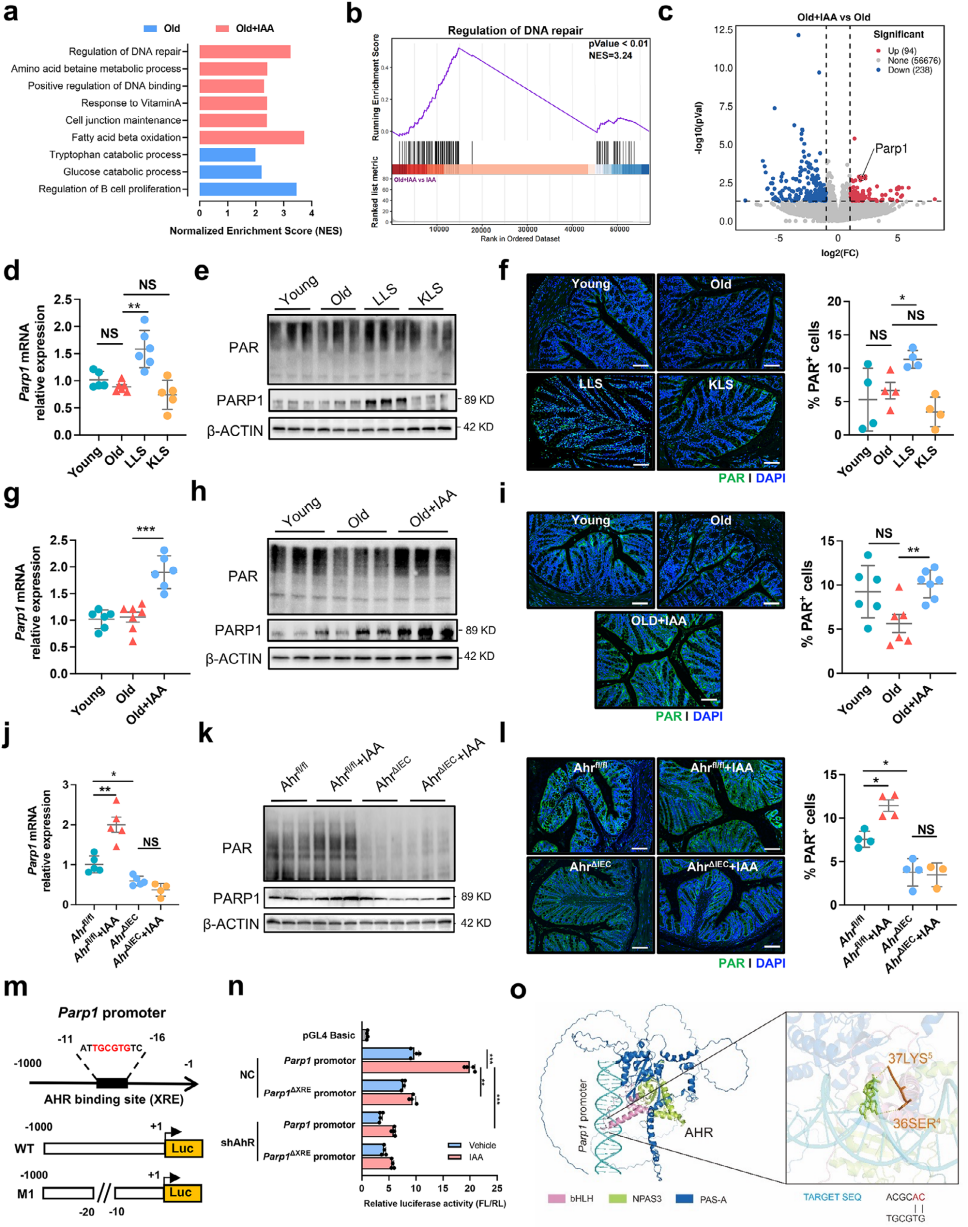
IAA supplementation mitigates intestinal aging via AHR‐PARP1 axis‐mediated DNA repair. a) Gene set enrichment analysis (GSEA) showing enrichment of DNA repair–related pathways in colonic tissues. b) GSEA enrichment plot for the Regulation of DNA repair pathway. c) Volcano plot of differentially expressed genes highlighting *Parp1*. d) Relative mRNA expression levels of *Parp1* in colon tissues of (*n* = 5 or 6 per group). e) Western blot analysis of PAR and PARP1 protein levels in colon tissues (*n* = 3 per group). f) Immunofluorescence staining of PAR (green) and DAPI (blue) in colonic sections (Scale bar = 100 µm). Quantification of PAR⁺ cells was shown on the right (*n* = 4 mice per group; three random fields per section). g) Relative *Parp1* mRNA expression in colon tissues from Old and Old + IAA groups measured by qRT‐PCR (n = 6 per group). h) Western blot analysis of PAR and PARP1 protein levels in colon tissues from Old and Old + IAA groups (*n* = 3 per group). i) Immunofluorescence staining of PAR (green) and DAPI (blue) in colonic sections (Scale bar = 100 µm). Quantification of PAR⁺ cells was shown on the right (*n* = 6 mice per group). j) Relative *Parp1* mRNA expression levels in *Ahr* ^fl/fl^ and *Ahr* ^ΔIEC^ mice treated with or without IAA (*n* = 5 per group). k) Western blot analysis of PARP1 protein levels in colon tissues of *Ahr* ^fl/fl^ and *Ahr* ^ΔIEC^ mice treated with or without IAA (*n* = 3 per group). l) Immunofluorescence staining of PAR (green) and DAPI (blue) in colonic sections (Scale bar = 50 µm). Quantification of PAR⁺ cells were shown on the right (n = 4 mice per group). m) Schematic illustration of the mouse Parp1 promoter region, showing the putative AhR binding site (XRE) and mutation design (M1). n) Luciferase reporter assay of *Parp1* promoter activity in cells transfected with either wild‐type or XRE‐mutated Parp1 promoter constructs, with or without IAA stimulation, in the presence or absence of AHR knockdown (shAHR). o) Molecular dynamics simulation of AHR binding to the *Parp1* promoter. Statistical significance was determined by one‐way ANOVA followed by Tukey's post hoc test. *P < 0.05; **P < 0.01; ***P < 0.001; NS, no significance.

To explore the potential mechanism of IAA‐mitigating intestinal aging, we performed transcriptomic profiling (RNA‐seq) of intestinal tissues from aged mice treated with and without IAA. Gene set enrichment analysis (GSEA) revealed that many pathways such as DNA regulation, fatty acid beta oxidation, and amino acid metabolism were enriched in aged mice upon IAA treatment (**Figure** **6**a). The DNA repair pathway was significantly upregulated following IAA supplementation (Figure 6a,b). Consistently, volcano plot analysis of differentially expressed genes showed that IAA treatment induced significant upregulation of 94 genes and downregulation of 238 genes in colon of aged mice (Figure 6c). Of particular note, gene *Parp1*, encoding a central enzyme mediating DNA repair for genomic integrity, was highlighted to be significantly upregulated in aged mice following IAA treatment (Figure 6c). RT‐qPCR and western blot analyses further confirmed that both mRNA and protein levels of PARP1 were markedly increased in aged mice upon supplementation with LLS, but not KLS, and IAA (Figure 6d,e). Notably, LLS instead of KLS significantly upregulated levels of DNA‐damage‐induced PARylation in intestine of aged mice (Figure 6e,f). IAA treatment also increased mRNA and protein levels of *Parp1* (Figure 6g,h) and promoted intestinal PARylation in aged mice (Figure 6h,i). However, IAA supplementation failed to upregulate *Parp1* expression and PARylation in intestine of *Ahr* ^ΔIEC^ mice (Figure 6j–l), indicating that IAA‐mediated *Parp1* induction is dependent on intestinal AHR signaling. Importantly, we discovered that a putative AHR response element (XRE) as one binding site of *Parp1* promoter from −1000 to −1 bp interacts with AHR protein via simulation of the JASPAR database (Figure 6m; Figure S10, Supporting Information). To experimentally verify AHR binding to *Parp1* promoter, we constructed a pGL4‐Basic‐*Parp1*‐promoter‐luc plasmid spanning from −1000 to −1 bp of *Parp1* promoter cloned upstream of luciferase in HEK 293T cells (Figure 6m). After transfection of *Parp1* luciferase reporter vectors into HEK 293T cells with and without AHR knockdown (Figure 6n), luciferase reporter assays demonstrated that IAA administration significantly enhanced luciferase activity of *Parp1* reporter, whereas no significant changes were observed in HEK 293T cells with both XRE mutation and *Ahr*‐knockdown upon IAA treatment (Figure 6n).

To further explore how AHR transcriptionally regulates DNA repair process, we performed molecular dynamics (MD) simulations to visualize the interaction between AHR and DNA sequence of *Parp1* promoter (Figure 6o). Structural prediction revealed that AHR complex forms a bHLH‐PAS heterodimer with NPAS3 and binds to the target DNA sequence of *Parp1* promoter via its basic helix‐loop‐helix (bHLH) and PAS‐A domains (Figure 6o). As shown in the model, PAS‐A domain of AHR (dark blue) and NPAS3 (light green) form a stable interface that facilitates docking onto the DNA helix. The binding site was mapped to a conserved XRE motif (5′‐TGCGTG‐3′), which is the canonical AHR response element. Notably, structural analysis highlighted a potential base‐specific interaction between residues Ser36 and Lys37 of bHLH and the target DNA sequence (ACGCAC/TGCGTG) of *Parp1* promoter (Figure 6o), suggesting direct base recognition at the binding site. These results provide structural support for a transcriptional regulatory role of AHR complex at XRE‐containing *Parp1* promoter.

### 2.6 AHR‐PARP1 Axis Regulates Cellular Senescence and DNA Repair in IECs

**Figure 7:**
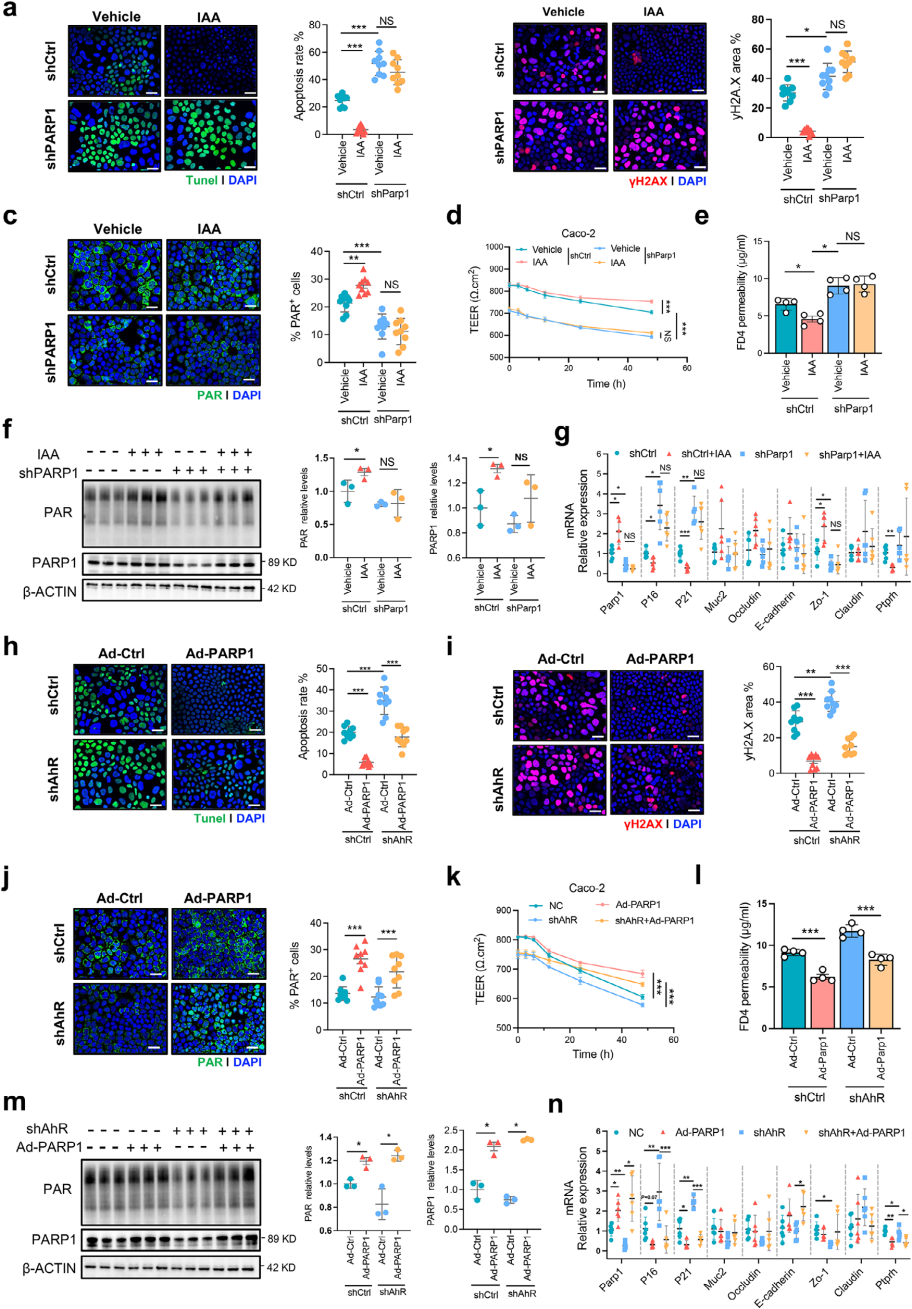
AHR‐PARP1 axis regulates DNA repair and cell senescence in vitro. a,h) TUNEL staining (green) and DAPI nuclear staining (blue), Scale bar = 50 µm. Quantification of apoptotic cells (TUNEL⁺) was shown on the right (mean ± SD, *n* = 3 per group; three random fields per section). b,i) Immunofluorescence staining of γH2A.X (red) and DAPI (blue), Scale bar = 50 µm. Quantification of γH2A.X⁺ area (% of total nuclear area) was shown on the right (mean ± SD, n = 3 per group; three random fields per section). c,j) Immunofluorescence staining of PAR (green) and DAPI (blue), Scale bar = 50 µm. Quantification of PAR⁺ area (% of total nuclear area) was shown on the right (mean ± SD, *n* = 3 per group; three random fields per section). d, k) Time‐course measurement of transepithelial electrical resistance (TEER) (*n* = 4 per group). e,l) Quantification of FD‐4 permeability (mean ± SD, *n* = 4 per group). f,m) Western blot analysis of PAR and PARP1 protein levels. β‐ACTIN served as a loading control. Quantification of PARP1 and PAR protein levels were shown on the right (mean ± SD, *n* = 3 per group). g,n) Relative mRNA expression levels of DNA repair‐ and barrier‐associated genes (mean ± SD, *n* = 6 per group). Statistical significance was determined by one‐way ANOVA followed by Tukey's post hoc test. *P < 0.05; **P < 0.01; ***P < 0.001; NS, no significance.

To investigate the requirement of *Parp1* in mediating DNA repair by IAA, we established senescent Caco‐2 cells with stable *Parp1* knockdown (shParp1) and overexpression (Ad‐Parp1) with and without IAA supplementation (Figure S9b,c, Supporting Information). TUNEL and γH2AX immunofluorescence staining combined with quantitative analyses showed that aged Caco‐2 cells with shParp1 exhibited higher levels of apoptosis and DNA damage, manifested by higher apoptosis rates and γH2AX‐positive areas than those in aged cells without shParp1, whereas IAA supplementation did not effectively downregulate their levels at least partly due to shParp1 (**Figure** **7**a,b). Of particular note, IAA treatment significantly increased proteins levels of PARP1 and promoted PARylation in senescent Caco‐2 cells, whereas no significant effects of IAA on proteins levels of PARP1 and PARylation in senescent Caco‐2 cells with shParp1 (Figure 7c,f). Furthermore, measurements of TEER and FD4 permeability suggested that IAA supplementation can restore epithelial barrier integrity in aged cells but failed to rescue TEER decline and increased gut permeability in aged cells with shParp1 (Figure 7d,e). RT‐qPCR analyses showed that IAA‐induced transcriptional restorations of intestinal barrier–related genes (*Zo‐1* and *Ptprh*) and senescence markers (*P16* and *P21*) in aged cell were abolished in shParp1 cells (Figure 7g). Similar to IAA treatment, *Parp1* overexpression (Ad‐Parp1) markedly restored apoptosis and DNA damage, shown with reduced apoptosis rate and γH2AX‐positive area in aged cell without shAHR (Figure 7h,i). Notably, Ad‐Parp1 also significantly upregulated levels of DNA‐damage‐induced PARylation in aged cells with and without shAHR (Figure 7j,m). Ad‐Parp1 also significantly increased membrane potential and decreased permeability of aged cell without shAHR (Figure 7k,l) accompanied with restoration of gut barrier and senescence‐related gene expression (Figure 7n). However, Ad‐Parp1 exhibited partial effects in ameliorating aforementioned gut barrier dysfunction and senescence phenotype of aged cell with shAHR (Figure 7h–n). These findings identify that AHR‐PARP1 axis regulates DNA repair process and cellular senescence in IECs.

## 3 Discussion

Progressive decline of organ function during aging, ultimately can trigger metabolic diseases such as osteoporosis, sarcopenia, cancer, and neurodegenerative pathologies.^[^ ^55^ ^]^ Among many organs, the gut is one of the most important tissues for host homeostasis due to its key physiological functions and crosstalk with other organs such as liver, bone, skeletal muscle, and brain.^[^ ^56^, ^57^, ^58^ ^]^ In this study, we found that both relatively old human and mice exhibit intestinal aging, manifested by significant gut barrier dysfunction and profound alterations of the gut microbiota and its metabolites. Notably, intestinal DNA damage and marked reduction in the relative abundance of *L. salivarius* and its metabolite IAA were also observed in humans and mice during aging. We proposed that supplementation with *L. salivarius* and/or IAA may ameliorate intestinal aging and aging‐related metabolic diseases. Upon investigating potential mechanisms by which *L. salivarius* and IAA supplementation ameliorate intestinal aging, it was found that AHR‐PARP1 signaling mediates a critical DNA repair process to counteract intestinal aging.

Our in vivo and in vitro results revealed for the first time that *L. salivarius* produces IAA from its precursor IAld via the bacterial enzyme ALDH rather than host cells. Consistent with prior studies, 16S rRNA profiling of the human cohort revealed an age‐related decline in the *Lactobacillus* genus, while murine metagenomics further identified reductions in several species, notably *L. salivarius*. We found LLS instead of KLS can effectively ameliorate aging‐induced intestinal barrier dysfunction, cell apoptosis and senescence and systemic inflammation. More importantly, *L. salivarius*‐derived IAA treatment also effectively ameliorated the aging phenotype in an intestinal AHR‐dependent manner, which was confirmed by genetic modifications with AHR in vivo and in vitro. Many probiotics such as *Roseburia intestinalis* and *Faecalibacterium prausnitzii* producing butyrate have been shown to alleviate neuropathic pain and the development of natural killer/T‐cell lymphoma (NKTCL).^[^ ^59^, ^60^, ^61^ ^]^ Fecal transplantation of *Lactobacillus reuteri* can ameliorate some metabolic diseases such as inflammatory bowel diseases (IBDs) and high fat diet‐induced obesity by regulating gut barrier function.^[^ ^62^ ^]^ Supplementation with microbial Trp metabolites such as IAA and IPA acting as endogenous AHR ligands was reported to effectively ameliorate osteoporosis via regulating intestinal AHR‐Wnt/𝛽‐catenin‐mediated gut‐bone axis.^[^ ^63^ ^]^ Previous studies have proposed that IAA has the capacity to enhance the efficacy of chemotherapy for pancreatic ductal adenocarcinoma (PDAC) by targeting ROS–autophagy axis.^[^ ^64^ ^]^ Commensal microbiota‐derived indole and IAA have been identified to extend lifespan and/or healthspan in *Drosophila* and mice by activating AHR‐Sirt2 signaling and inhibiting downstream mTOR pathway.^[^ ^65^ ^]^ Collectively, these results highlight that AHR plays critical roles in maintaining host homeostasis and probiotics and its metabolites (e.g., *L. salivarius* and IAA) hold promises as potential interventions for aging and aging‐related diseases. However, the relative contribution of local colonic versus systemic actions remains to be clarified since orally administered IAA is largely absorbed systemically. Here higher levels of IAA in intestine than that in liver of mice when oral exposure at different dosages suggested that IAA has anti‐intestinal aging effects, which are of high relevance to commensal bacteria. Future validation studies employing the LLS versus KLS comparison or targeted colonic delivery of IAA will help to substantiate the causal role of the aging‐associated microbiota.

Theoretically, the rate of aging is determined by the ratio between accumulation of damage and compensation/repair mechanisms. During aging, DNA damage escapes the control of the compensation system, thus resulting in phenotypic aging.^[^ ^43^ ^]^ In this study, we found that *L. salivarius*‐derived IAA was responsible for repairing intestinal DNA damage of both aged mice and senescent cells by activating AHR. Mechanistically, AHR activation by IAA supplementation effectively mitigated intestinal aging‐related barrier function by stimulating PARP1‐mediated DNA repair and polymerization of poly (ADP‐ribose) (PARylation). Of particular note, IAA supplementation promoted direct regulation of *Parp1* expression by facilitating AHR binding to its promoter region. Structurally, bHLH and PAS‐A domains of AHR upon IAA combined with DNA sequence of *Parp1* promoter formed a stable complex, thus leading to enhancement of PARP1 activity and consequent DNA repair. These results suggest that the AHR‐PARP1 axis is crucial for alleviating IESc senescence by maintaining genome stability.

PARP1 is a well‐characterized DNA damage sensor and repair enzyme that binds to DNA lesions and promotes PARylation, which serves as a scaffold for recruitment of DNA repair machinery.^[^ ^47^ ^]^ Here we found that IAA supplementation significantly upregulated expression of PARP1 and promoted PARylation in an intestinal AHR‐dependent manner that were confirmed by both in vivo and in vitro *Ahr*‐deficient models, thereby repairing DNA damage. Consistently, PARP1 activation has been shown to counteract obesity‐induced adipocyte senescence through SREBP1c–PARP1 signaling axis.^[^ ^66^ ^]^ Moreover, PARP1 is highly expressed in several long‐lived species, such as the naked mole‐rat, where it contributes to efficient DNA repair and genomic maintenance, thereby promoting longevity.^[^ ^67^ ^]^ Elevated PARP1 activity has also been observed in the cells of human centenarians, suggesting a possible role in lifespan extension.^[^ ^68^, ^69^ ^]^ However, other studies suggested a context‐dependent detrimental effect of PARP1 overactivation. For instance, excessive PARP1 activity shortens lifespan of *Drosophila*, while its deletion prolongs survival and improves mitochondrial homeostasis through AMPK signaling pathway.^[^ ^70^ ^]^ Similarly, increased PARP1 activity has been reported in aged human tissues, including cerebrovascular endothelial cells, where chronic activation contributes to NAD⁺ depletion and blood–brain barrier (BBB) dysfunction.^[^ ^50^ ^]^ Pharmacological inhibition of PARP1 in these models restored intracellular NAD⁺ levels and improved BBB integrity, offering protection against age‐related brain decline.^[^ ^50^ ^]^ Collectively, these findings suggest that PARP1 exhibits a dual role in aging. Under homeostatic conditions, moderate activation of PARP1 facilitates rapid DNA repair through PARylation, helping to preserve genomic stability and cellular viability in aging tissues. In contrast, chronic PARP1 overactivation leads to excessive NAD⁺ consumption, suppression of longevity‐associated sirtuins (e.g., SIRT1 and SIRT3), and metabolic collapse, forming a vicious cycle of “DNA damage–PARP1 hyperactivation–excessive NAD⁺ depletion–metabolic dysfunction” that accelerates cellular senescence and apoptosis.^[^ ^71^, ^72^ ^]^ Therefore, although our findings support beneficial roles of AHR activation‐enhanced PARP1 signaling in maintaining intestinal homeostasis during aging, these controversial effects of sustained PARP1 activity on DNA repair and host metabolism warrant further exploration.

## 4 Conclusion

In this study, we identified for the first time that IAA acting as one of AHR endogenous ligands is derived from *Lactobacillus salivarius* rather than host cells. Furthermore, we discovered a functional role of AHR upon IAA supplementation in alleviating intestinal aging by facilitating PARP1‐mediated NDA repair in aged mice and cells. Therefore, we would like to propose AHR‐PARP1 axis for DNA repair to counteract aging and age‐related disorders. Although activation of PARP1 expression can compensate DNA damage responses and senescent phenotype of aged mice and cells to a certain degree, the specific cell types and organelles in the gut where AHR‐PARP1 axis exerts its functions for DNA repair remain unclear. Further study is needed to elucidate the precise mechanisms by which microbiota‐AHR‐PARP1 axis contribute to intestinal aging and aging‐related dysfunction.

## 5 Experimental Section

### Human Cohort Study

The human cohort studies were approved by the ethics committee of AIage Life Science Corporation (ref.no.2019‐001) and the First Affiliated Hospital of Guangxi Medical University (ref.no.2020‐KT‐050). All participants signed an informed consent agreement before donating their fecal samples. Feces of healthy individuals was collected and categorized into Young (20–44 years old, *n* = 314) and Old (66–85 years old, n = 386) group. Exclusion criteria included: history of chronic gastrointestinal, metabolic, inflammatory, or autoimmune diseases; current malignancy; use of antibiotics, probiotics, prebiotics, or immunosuppressive agents within the past 3 months; recent hospitalization or surgery; and incomplete demographic or clinical information. Participants with acute infection or unwillingness to provide stool samples were also excluded. For all subjects, fasting was required for 12 h prior to sampling. The fecal samples were used for microbiome and metabolome analyses.

### In Vitro Experiments

*Lactobacillus salivarius* was purchased from DSMZ (#20 555) and cultured in de Man, Rogosa and Sharpe (MRS) broth under anaerobic conditions for 24 h. After incubation, cultures were washed with PBS and concentrated in anaerobic PBS containing 25% (vol/vol) glycerol to a final concentration of ≈1 × 10^10^ CFU mL^−1^. The bacterial stocks were stored at −80 °C until use. To assess viability, serial dilutions in PBS were plated on MRS agar and incubated anaerobically for 48 h to determine colony‐forming units (CFU mL^−1^). To assess whether *Lactobacillus salivarius* could metabolize tryptophan into indole‐3‐acetaldehyde (IAA) via aldehyde dehydrogenase (ALDH) in vitro, bacteria were cultured in MRS medium alone (Ctrl) and treated with *L. salivarius* alone (LS), and *L. salivarius* + ALDH inhibitor KS106 at 1 µm (LSK). After incubation, culture supernatants were collected by centrifugation at 4000 g for 10 min and filtered through a 0.22 µm membrane to remove bacterial cells. The concentration of IAA in the supernatants was then measured using liquid chromatography–mass spectrometry (LC‐MS). To confirm that KS106 treatment did not affect bacterial growth, *L. salivarius* was cultured in MRS broth containing various concentrations of KS106 (0, 1, 10, 100, and 1000 nm), and optical density at 600 nm (OD600) was recorded over time to monitor proliferation.

### In Vivo Experiments

All animal experimental procedures were approved by the animal ethics committee of Innovation Academy for Precision Measurement Science and Technology, CAS (APM No: APM20029, China). Aged and young male C57BL/6J mice (Wukong Biotechnology, Jiangsu, China), *Ahr* ^flox/flox^ and intestine‐specific *Ahr* knockout (*Ahr* ^ΔIEC^) male C57BL/6J mice (Sai Ye Biotechnology, Jiangsu, China) were housed under specific pathogen‐free (SPF) conditions with a 12‐h light‐dark cycle and fed sterilized food with autoclaved water adlibitum. Each animal was checked for its suitability according to animal welfare authorities before individual experiment.

Animal experiment 1: Microbial and metabolic profiling in feces of young and aged mice. Fecal samples were collected from young (2–3 months old, *n* = 8 per group) and old (19–20 months old, *n* = 8) C57BL/6J mice. Fecal pellets were freshly collected in sterile tubes, snap‐frozen in liquid nitrogen, and stored at −80 °C until analysis. DNA and metabolites were extracted from the fecal samples for microbial analysis and tryptophan metabolites quantification.
Animal experiment 2: Antibiotic treatment and *L. salivarius* colonization. Male C57BL/6J mice (2–3 months old; n = 6 per group) were first acclimated for one week and then randomly assigned to three groups. The vehicle group received daily oral gavage of PBS for two weeks. The ABX group was treated with a broad‐spectrum antibiotic cocktail (ABX: 1 mg mL^−1^ ampicillin, 0.5 mg mL^−1^ vancomycin, 1 mg mL^−1^ metronidazole, and 1 mg mL^−1^ neomycin) dissolved in anaerobic PBS for two weeks. The ABX + *L. salivarius* group received the same ABX treatment during the first week, followed by daily oral gavage of *L. salivarius* during the second week. At the end of the two‐week treatment period, mice were sacrificed, and fecal and intestine samples were collected for further analysis.
Animal experiment 3: *L. salivarius* colonization and IAA production. Male C57BL/6J mice (2–3 months old; *n* = 6 per group) were orally administered *L. salivarius* DSM 20 555 (10⁸ CFU mL^−1^ kg^−1^ body weight, suspended in PBS) daily for 7 consecutive days. Fecal samples were collected at baseline (day –1) and after treatment (day 7). The relative abundance of *L. salivarius* was quantified by qPCR using species‐specific primers and fecal IAA concentrations were measured by liquid chromatography–mass spectrometry (LC–MS/MS) to evaluate microbial tryptophan metabolism in vivo.
Animal experiment 4: Effects of live or heat‐killed *L. salivarius* on aged mice. Aged C57BL/6J mice (18–19 months old, *n* = 8 per group) were randomly assigned to receive either PBS (Old), live *L. salivarius* (LLS), or heat‐killed *L. salivarius* (KLS) via oral gavage three times per week for one month. The bacterial suspension was prepared in PBS (10⁸ CFU mL^−1^ kg^−1^). Correspondently, young mice (2–3 months old, *n* = 8) serving as the controls (Young) were received PBS for one month.
Animal experiment 5: IAA supplementation to wild type mice. Aged C57BL/6J mice (18–19 months, *n* = 8 per group) were assigned to receive either vehicle (Old) or IAA (50 mg kg^−1^) dissolved in corn oil via oral gavage three times per week for two months. Young mice (2–3 months old, *n* = 8) serving as controls (Young) were received an equal volume of corn oil vehicle for two months.
Animal experiment 6: IAA supplementation to aged mice with and without intestine‐specific *Ahr* knockout. Aged *Ahr* ^flox/flox^ and *Ahr* ^ΔIEC^ mice (18–19 months old, n = 8 per group) were orally administered with and without IAA (50 mg/kg) dissolved in corn oil or corn oil (Vehicle)three times per week for two months.

At the end of each experiment, mice were fasted overnight and then euthanized under deep anesthesia using intraperitoneal injection of pentobarbital sodium (100 mg kg^−1^), followed by cervical dislocation. Blood, intestinal contents, and tissue samples were collected immediately after euthanasia. Samples were snap‐frozen in liquid nitrogen and stored at –80 °C until further analysis.

### Cell Culture and Treatments

HEK293T cells (#GDC0187) and Caco‐2 cells (#GDC0153) were obtained from the China Center for Type Culture Collection (CCTCC, Wuhan University, Wuhan, China). HEK293T cells were cultured in Dulbecco's Modified Eagle's Medium (DMEM; Gibco, USA) supplemented with 10% fetal bovine serum (FBS; Gibco, USA) and 1% penicillin‐streptomycin (Gibco, USA). Caco‐2 cells were maintained in Minimum Essential Medium (MEM; Gibco, USA) supplemented with 20% FBS and 1% penicillin‐streptomycin at 37 °C in a humidified atmosphere of 5% CO_2_. To induce different cellular states, normal Caco‐2 cells were treated under the following conditions: Quiescent cells (QUI): Cells were cultured in medium containing 0.2% FBS for 3 days. Senescent cells (SEN): Cells were treated with 250 nm doxorubicin (DOXO; Sigma‐Aldrich) for 7 days. For IAA treatment, IAA was added to the culture medium starting from day 5 of DOXO treatment and maintained until day 7 for subsequent experimental analyses. Cells were monitored morphologically, and the induction of quiescence and senescence was confirmed with subsequent assays. To investigate the ability of Caco‐2 cells to metabolize IALD (indole‐3‐acetaldehyde) into IAA (indole‐3‐acetic acid), cells were seeded in 12‐well plates and cultured until ≈80% confluence. Cells were then incubated with 100 µm IALD (Sigma‐Aldrich) for 6 h. Culture supernatants were collected before and after treatment, and the concentration of IAA was quantified using LC‐MS analysis. To evaluate the capacity of Caco‐2 cells for de novo IAA biosynthesis, cells were treated with 100 µm ^13^C‐labeled tryptophan (^13^C‐tryptophan; Cambridge Isotope Laboratories) for 12 h. After incubation, culture media were harvested and subjected to LC‐MS to detect 13C‐labeled IAA. The incorporation of ^13^C into IAA was confirmed by comparing the mass spectra and retention times of labeled and unlabeled metabolites.

### Generation of Stable Cell Lines

Lentiviral particles encoding non‐targeting shRNA as normal control (NC) and AHR‐specific shRNA (shAHR) were generated by co‐transfecting 293T packaging cells with pLKO.1‐based shRNA constructs (Integrated Biotech Solutions Co., Ltd, IBS), along with the packaging plasmid pR8.2 and the envelope plasmid pMDG.2, using Lipofectamine 3000 (Invitrogen, USA). Separately, lentiviral particles for PARP1 overexpression (Ad‐PARP1) were generated using a pLVX‐based expression vector (Integrated Biotech Solutions Co., Ltd, IBS) with the same packaging system. After 18 h of transfection, the culture medium was replaced, and cells were incubated for an additional 48 h. Viral supernatants were collected, filtered through a 0.45‐µm filter, and used to infect Caco‐2 cells in the presence of 5 µg mL^−1^ Polybrene (Sigma‐Aldrich) to enhance transduction efficiency. Following 72–96 h of infection, cells were selected with 3 µg mL^−1^ puromycin and maintained in 1.5 µg mL^−1^ puromycin thereafter. Knockdown efficiency of AHR and overexpression of PARP1 were confirmed by quantitative reverse‐transcription PCR (qRT‐PCR) and Western blotting.

### 5.1 16S rRNA Gene Sequencing and Metagenomic Sequencing

Fecal DNA was extracted from human and mouse stools using a soil DNA Kit and subjected to sequencing using Illumina NovaSeq 6000 platform (Majorbio Bio‐pharm Technology Co., Ltd.). The V3–V4 regions of 16S rRNA genes were amplified using a 6universal primer. Denoising was performed on the sequences obtained to trim low‐quality reads, eliminate chimera, and identify amplicon sequence variants (ASVs) using Divisive Amplicon Denoising Algorithm 2 (DADA2) on QIIME2 (version 2021.11). Taxonomic classification was conducted using the SILVA database (version 138.1) for microbial annotation. Metagenomic sequencing was conducted using the Illumina platform (Majorbio Bio‐pharm Technology Co., Ltd.). Genomic DNA was extracted from fecal samples and quality was assessed using 1% agarose gel electrophoresis. Qualified DNA was then randomly fragmented to ≈350 bp using a Covaris M220 instrument. Paired‐end (PE) sequencing libraries were constructed by ligating Y‐shaped adapters, followed by magnetic bead purification to remove unligated fragments. Libraries were enriched by PCR amplification and subsequently denatured to generate single‐stranded DNA. Bridge PCR was utilized to form DNA clusters on the flow cell, where one end of the DNA fragment was hybridized to complementary primers immobilized on the chip surface, while the other end attached nearby, forming bridge‐like structures. After amplification to generate clusters and subsequent linearization into single strands, sequencing was performed by cyclic addition of fluorescently labeled nucleotides, laser excitation, and image capture, ultimately yielding the sequencing data for downstream bioinformatic analyses. Genes obtained from each sample were grouped, and redundant sequences were eliminated by clustering with CD‐HIT (v4.8.1), applying thresholds of > 90% coverage and > 95% identity. The expression levels for individual genes across samples were quantified using Salmon (v1.10.1) and expressed as TPM (transcripts per million). These TPM values served as indicators of relative gene abundance, and were subsequently summed to determine the relative abundances of species and functional categories. Based on the KEGG database, further functional profiling of the metagenomic sequencing data was conducted.

### RNA Sequencing and Data Analysis

Total RNA was extracted from mouse colon tissues using the TRIzol reagent (Invitrogen, USA). RNA quality and integrity were assessed using a NanoDrop spectrophotometer and an Agilent 2100 Bioanalyzer (Agilent Technologies, USA). Only samples with an RNA integrity number (RIN) ≥ 7.0 were used for library construction. RNA sequencing was performed by Majorbio Bio‐pharm Technology Co., Ltd. (Shanghai, China). Three biological replicates were included for each group (Old and Old + IAA). Sequencing libraries were prepared using the NEBNext Ultra RNA Library Prep Kit for Illumina (NEB, USA) and sequenced on an Illumina platform to generate paired‐end 150 bp reads. Raw reads were quality‐filtered using Trimmomatic and mapped to the mouse reference genome (GRCm38/mm10) using STAR aligner. Gene levels were quantified using feature Counts and normalized as fragments per kilobase of exon model per million mapped fragments (FPKM). Differentially expressed genes (DEGs) between the Old and Old + IAA groups were identified using the DESeq2 package in R, with thresholds set at adjusted *p* < 0.05 and |log_2_ fold change| > 1. Gene Set Enrichment Analysis (GSEA) was conducted using software (Broad Institute, USA) with the hallmark gene sets from the Molecular Signatures Database (MSigDB). Normalized enrichment score (NES), nominal p‐values, and false discovery rate (FDR) were calculated for pathway enrichment assessment.

### Target Quantification of Tryptophan Metabolites

Tryptophan metabolites were extracted from fecal samples (10 mg per sample) and serum (10 µL per sample) using a mixture of methanol and acetonitrile: water (1:1, v/v) containing 0.1% formic acid, with d5‐tryptophan as the internal standard. Samples were homogenized using a Qiagen Tissue‐Lyser at 20 Hz for 90 s, followed by two rounds of extraction and centrifugation. Supernatants were combined, dried under vacuum, and reconstituted in acetonitrile:water (1:1, v/v) with 0.1% formic acid. Metabolite profiling was conducted on an Agilent 1290 UHPLC system coupled to a 6460 triple quadrupole mass spectrometer. Metabolites were identified and quantified using via HPLC‐QQQ‐MS/MS with multiple reaction monitoring (MRM).

### Histopathology

Intestinal tissues were collected from euthanized mice, rinsed with cold PBS, and fixed in 4% paraformaldehyde at 4 °C for 24 h. Samples were then dehydrated through a graded ethanol series, cleared in xylene, embedded in paraffin, and sectioned at 4 µm thickness. Paraffin‐embedded intestinal sections were deparaffinized, rehydrated, and stained with hematoxylin and eosin (H&E) to assess epithelial architecture. Slides were dehydrated, mounted, and visualized under a bright‐field microscope at 20× magnification. To assess mucus production and goblet cell abundance, intestinal sections were stained with Alcian Blue–Periodic Acid–Schiff (AB‐PAS). After deparaffinization and rehydration, sections were stained with Alcian Blue (pH 2.5) for acidic mucins, oxidized with periodic acid, and incubated with Schiff's reagent to stain neutral mucins. Hematoxylin was used for nuclear counterstaining. Slides were imaged at 4× and 20× magnification. For quantitative analysis, three non‐overlapping fields per section at 20 × magnification were randomly selected. The number of goblet cells per crypt was manually counted using ImageJ software (NIH, Bethesda, MD, USA), and mucus layer thickness was measured at five evenly spaced points per field using the straight‐line tool in ImageJ. The mean values per mouse were calculated for statistical comparisons.

### TUNEL Staining

TUNEL (Terminal deoxynucleotidyl transferase dUTP Nick‐End Labeling) staining was performed using a One‐Step TUNEL Apoptosis Assay Kit (Beyotime, C1090) according to the manufacturer's instructions. For colon sections, paraffin‐embedded tissues were deparaffinized, rehydrated through graded ethanol series, and permeabilized with proteinase K at room temperature. After washing with PBS, sections were incubated with the TUNEL reaction mixture at 37 °C for 1 h in a humidified chamber. Nuclei were counterstained with DAPI (blue fluorescence). Samples were mounted with antifade medium and imaged using a confocal laser scanning microscope. TUNEL‐positive signals were detected as green fluorescence (excitation at 488 nm), and nuclei were visualized by DAPI staining under the blue channel. For Caco‐2 cells, cells were fixed with 4% paraformaldehyde for 30 min at room temperature, permeabilized with 0.1% Triton X‐100 in PBS for 5 min, and incubated with the TUNEL reaction mixture at 37 °C for 1 h in the dark. After washing, cells were counterstained with DAPI. Fluorescence images were acquired using confocal microscopy, with TUNEL signals captured in the green channel (488 nm) and nuclei visualized by DAPI staining in the blue channel.

### Immunofluorescence Staining

Immunofluorescence staining was performed on paraffin‐embedded mouse colon sections and Caco‐2 cell coverslips. For colon tissue, fresh samples were fixed in 4% paraformaldehyde for 24 h, dehydrated, paraffin‐embedded, and sectioned at 3–4 µm thickness. After deparaffinization, rehydration, and antigen retrieval, sections were blocked with 5% BSA in PBS for 1 h at room temperature and incubated overnight at 4 °C with anti‐γH2A.X primary antibody (1:500, Affinity), anti‐ZO‐1 antibody (1:1000, Proteintech) or PAR antibody (1:4000, Cell Signaling Technology). Following PBS washing, sections were incubated with Alexa Fluor 488–conjugated secondary antibody (1:1000, ThermoFisher) for 1 h at room temperature in the dark. Nuclei were counterstained with DAPI (Solarbio). Slides were mounted with antifade mounting medium, and images were acquired using a Nikon A1 confocal laser scanning microscope with a 20× objective. γH2A.X and PAR signals were visualized in green (488 nm) and nuclei in blue. For Caco‐2 cells, cells cultured on coverslips were fixed with 4% paraformaldehyde for 15 min at room temperature, permeabilized with 0.1% TritonX‐100 in PBS for 5 min, and blocked with 5% BSA. Cells were then incubated overnight at 4 °C with anti‐γH2A.X primary antibody, anti‐ZO‐1 antibody or PAR antibody, followed by Alexa Fluor 488–conjugated secondary antibody or Alexa Fluor 594–conjugated secondary antibody (1:1000, ThermoFisher) for 1 h in the dark. After washing, nuclei were counterstained with DAPI. Fluorescence images were obtained using a Nikon A1 confocal microscope with a 40× objective. γH2A.X signals were visualized in green (488nm) or red (594 nm) and nuclei in blue. Quantification of γH2A.X‐positive cells in both colon tissue sections and Caco‐2 cell coverslips was performed using ImageJ software (NIH, Bethesda, MD, USA). For each sample, three randomly selected fields were captured under identical exposure settings using a Nikon A1 confocal microscope with a 40× objective. γH2A.X‐positive nuclei and total DAPI‐stained nuclei (blue fluorescence) were manually counted using the Cell Counter plugin in ImageJ. The percentage of γH2A.X‐positive cells was calculated as the number of γH2A.X‐positive nuclei divided by the total number of nuclei. The mean value from three fields was used to represent each sample.

### Enzyme‐Linked Immunosorbent Assay

Enzyme‐linked immunosorbent assays were performed to quantify the levels of pro‐inflammatory cytokines (IL‐6, IL‐1β, and TNF‐α) and lipopolysaccharide (LPS) in mouse serum and colonic tissue. Tissue samples were first homogenized in cold PBS containing protease inhibitors and then centrifuged at 12000 × g for 10 min at 4 °C to collect the supernatant. Serum samples were prepared by allowing whole blood to clot at room temperature for 30 min, followed by centrifugation at 3000 × g for 10 min. Quantification of analytes was carried out using mouse‐specific ELISA kits (Shanghai Huyu Biotechnology Co., Ltd., China) according to the manufacturer's instructions. Briefly, 96‐well plates pre‐coated with target‐specific antibodies were incubated with standards and diluted samples at room temperature for 1–2 h. After a series of washing steps, HRP‐conjugated detection antibodies were added, followed by incubation with TMB substrate solution. Color development was stopped with 2 M sulfuric acid, and absorbance was measured at 450 nm using a microplate reader (BioTek, USA). All measurements were performed in duplicate, and cytokine or LPS concentrations were calculated based on standard curves generated for each analyte.

### Quantitative Real‐Time PCR

Total RNA was extracted from mouse colon tissues and Caco‐2 cells using TRIzol reagent (Invitrogen, USA) according to the manufacturer's instructions. RNA concentration and purity were determined by NanoDrop spectrophotometry. cDNA was synthesized from 1 µg of total RNA using a reverse transcription kit (Takara, Japan) following the provided protocol. Quantitative real‐time PCR was performed using SYBR Green PCR Master Mix (Takara, Japan) on a CFX96 Touch Real‐Time PCR Detection System (Bio‐Rad, USA). The thermocycling conditions were 95 °C for 30 s, followed by 40 cycles of 95 °C for 5 s and 60 °C for 30 s. All reactions were run in triplicate. Gene expression was normalized to β‐actin, and relative expression levels were calculated using the 2^^–ΔΔCt^ method. The primer sequences used for mouse (m) and human (h) genes are listed in Table S1 (Supporting Information).

### Western Blotting

Proteins were extracted from mouse colon tissues or Caco‐2 cells using RIPA lysis buffer supplemented with protease inhibitors (Beyotime, China). Protein concentrations were determined using a BCA Protein Assay Kit (Beyotime, China) following the manufacturer's instructions. Equal amounts of protein (20–30 µg) were separated by SDS‐PAGE and transferred onto PVDF membranes (Millipore, USA). Membranes were blocked with 5% non‐fat milk in TBST for 1 h at room temperature and incubated overnight at 4 °C with the following primary antibodies: anti‐β‐actin (1:50000; Proteintech), anti‐Parp1 (1:4000; Proteintech) and anti‐PAR (1:500, Cell Signaling Technology). After washing, membranes were incubated with HRP‐conjugated secondary antibodies for 1 h at room temperature: anti‐mouse IgG (1:5000; Proteintech) or anti‐rabbit IgG (1:5000; Proteintech), depending on the host species of the primary antibody. Protein bands were visualized using an enhanced chemiluminescence (ECL) detection system (Thermo Fisher Scientific, USA) and imaged with a ChemiDoc XRS+ System (Bio‐Rad, USA). Densitometric analysis was performed using ImageJ software.

### Transepithelial Electrical Resistance Measurement and Cellular Permeability Assay

Caco‐2 cells (1 × 10⁵ cells mL^−1^) were seeded into the upper chambers of Transwell inserts (polyester membrane, 0.4 µm pore size; Corning, USA) and cultured for ≈21 days to allow full differentiation and monolayer formation. Transepithelial electrical resistance (TEER) was measured using a Millicell‐ERS voltohmmeter (Millipore, USA) to evaluate monolayer integrity. Only monolayers with TEER values exceeding 500 Ω·cm^2^ were used for permeability assays. For the paracellular permeability assay, fluorescein isothiocyanate–labeled dextran (FD‐4; 1 mg mL^−1^ in HBSS) was added to the apical chamber (100 µL). After incubation at 37 °C for 30 min, 100 µL of medium from the basolateral chamber was collected and transferred into a black 96‐well plate. Fluorescence intensity was measured at an excitation wavelength of 480 nm and an emission wavelength of 520 nm using a microplate reader (BioTek, USA). The amount of FD‐4 that permeated across the cell monolayer was used to assess paracellular permeability.

### Dual‐Luciferase Reporter Assay

The human PARP1 promoter sequence (−1000 to −1 bp relative to the transcription start site) was amplified from human genomic DNA and cloned into the pGL4.10 vector (Promega) upstream of the firefly luciferase reporter gene to generate the PARP1 promoter luciferase construct. For mutation of the AHR response element (XRE) site, site‐directed mutagenesis was performed using the QuikChange Lightning kit (Agilent Technologies) according to the manufacturer's instructions. HEK293T cells were seeded in 24‐well plates and transfected at ≈70% confluence with 400 ng of the reporter plasmid and 40 ng of pRL‐TK Renilla luciferase plasmid (Promega) as an internal control, using Lipofectamine 3000 (Invitrogen). Where indicated, cells were co‐transfected with AHR‐targeting shRNA (shAHR) or control shRNA plasmids. After 24 h, cells were treated with or without IAA (100 µm) for an additional 24 h. Firefly and Renilla luciferase activities were measured sequentially using the Dual‐Luciferase Reporter Assay System (Promega) on a GloMax 20/20 luminometer (Promega) according to the manufacturer's protocol. Firefly luciferase activity was normalized to Renilla luciferase activity for each sample, and results were expressed as relative luciferase units (RLU). All experiments were performed in triplicate.

### Molecular Dynamics (MD) Simulations

Molecular dynamics (MD) simulations were performed to investigate the binding interactions between AHR and the *Parp1* promoter DNA. All MD simulations were carried out using the CHARMM36 force field and implemented via the CHARMM‐GUI platform. The TIP3P water model was applied for solvation, and periodic boundary conditions were used in conjunction with particle mesh Ewald (PME) electrostatics, with a PME grid spacing of ≈1 Å per grid point. The AHR–DNA complex was solvated using the Solvate tool, which placed water molecules around the complex in energetically favorable conformations. Potassium and chloride ions were randomly substituted for water molecules to achieve a final ionic strength of 100 mm. The simulations were conducted under an NPT ensemble (constant number of atoms, pressure, and temperature), with the temperature maintained at 291 K. An initial energy minimization was followed by equilibration runs without restraints. The system was then subjected to production MD simulations for ≈200 ns. A stable configuration representing the equilibrated state was extracted after ≈100 ns for subsequent analysis. The structure of AHR protein was generated by homology modeling based on AlphaFold2‐predicted models. The 3D conformation of the DNA sequence corresponding to *Parp1* promoter was obtained from the AlphaFold Server (https://alphafoldserver.com/).

### Statistical Data Analysis

Statistical data analysis was performed using GraphPad Prism software (version 8.0). Correlation network analyses were conducted using R software (version 4.2.1). Data were presented as mean ± standard deviation (SD). An unpaired two‐tailed Student's t‐test was used for comparisons between two groups. For comparisons among multiple groups, one‐way ANOVA followed by Tukey's post hoc test was performed. P‐values < 0.05 were considered statistically significant.

## Conflict of Interest

The authors declare no conflict of interest.

## Author Contributions

Z.C. and C.Z. contributed equally to this work. G.C., Z.L.M., and A.D.P. designed the projects and supervised the experiments. Z.C., C.Z., and H.L. performed the experiments. W.L., W.Y., X.G., Y.H., X.L., Q.X., and Z.Z. participated in the data analysis. Q.X., Z.Z., and W.L. helped to collect the clinical samples and provided helpful suggestions for the project and participated in the discussion. Z.C. wrote the draft. Z.L.M. and A.D.P reviewed and edited the manuscript.

## Supporting information



Supporting Information

## Data Availability

The gut microbiota sequencing data of mice (PRJNA1344461) and humans (PRJNA830660) and RNA sequencing data of mice (PRJNA1344669) have been deposited in the National Center for Biotechnology Information GenBank repository. The datasets generated during the current study are available from the corresponding author on reasonable request.
