# Supplementary material for: Lactobacillus Salivarius‐Derived Indole‐3‐Acetic Acid Promotes AHR‐PARP1 Axis‐Mediated DNA Repair to Mitigate Intestinal Aging
Source: Adv Sci (Weinh). 2025 Oct 28;13(4):e15794. doi: 10.1002/advs.202515794 (PMC12822427; doi:10.1002/advs.202515794)
Supplement: Supplementary file 1 — Supporting Information [file ADVS-13-e15794-s001.docx]

**Supplementary information**

***Lactobacillus salivarius*-derived indole****-3-acetic acid promotes AHR-PARP1 axis-mediated DNA repair to** **mitigate intestinal aging**

Zheng Cao,^1,2,7^ Cui Zhang,^1,2,7^ Hehua Lei,^1,2^ Weichuan Lin,^1^ Wenkai Yu,^1,2^ Xin Gao,^1^ Yanmeng He,^1,2^ Xinzhi Li,^3^ Qingwei Xiang,^4^ Zhiwen Zhang,^4^ Weifei Luo,^5^ Andrew D. Patterson,^6,*^ Limin Zhang,^1,2,*^ Gang Chen^4,*^

^1^State Key Laboratory of Magnetic Resonance and Imaging, National Centre for Magnetic Resonance in Wuhan, Innovation Academy of Precision Measurement Science and Technology, Chinese Academy of Sciences (CAS), Wuhan 430071, China;

^2^University of Chinese Academy of Sciences, Beijing 100049, China;

^3^School of Pharmacy, Faculty of Medicine, Laboratory for Drug Discovery from Natural Resource, State Key Laboratory of Quality Research in Chinese Medicine, Macau University of Science and Technology, Macao 999078, China;

^4^Hubei Shizhen Laboratory, Department of Geriatrics & Department of Orthopedic Surgery, Hubei Provincial Hospital of Traditional Chinese Medicine (Affiliated Hospital of Hubei University of Chinese Medicine), Wuhan 430060, China;

^5^Guangxi Key Laboratory of Longevity Science and Technology, AIage Life Science Corporation Ltd., Nanning 530200, China;

^6^Department of Veterinary and Biomedical Sciences, The Pennsylvania State University, University, Park, Pennsylvania, USA;

^7^These authors contributed to this work equally.

*Correspondence: zhanglm@wipm.ac.cn (L. Zhang), chengang12@hbucm.edu.cn (G. Chen) and adp117@psu.edu (A.D. Patterson)

**
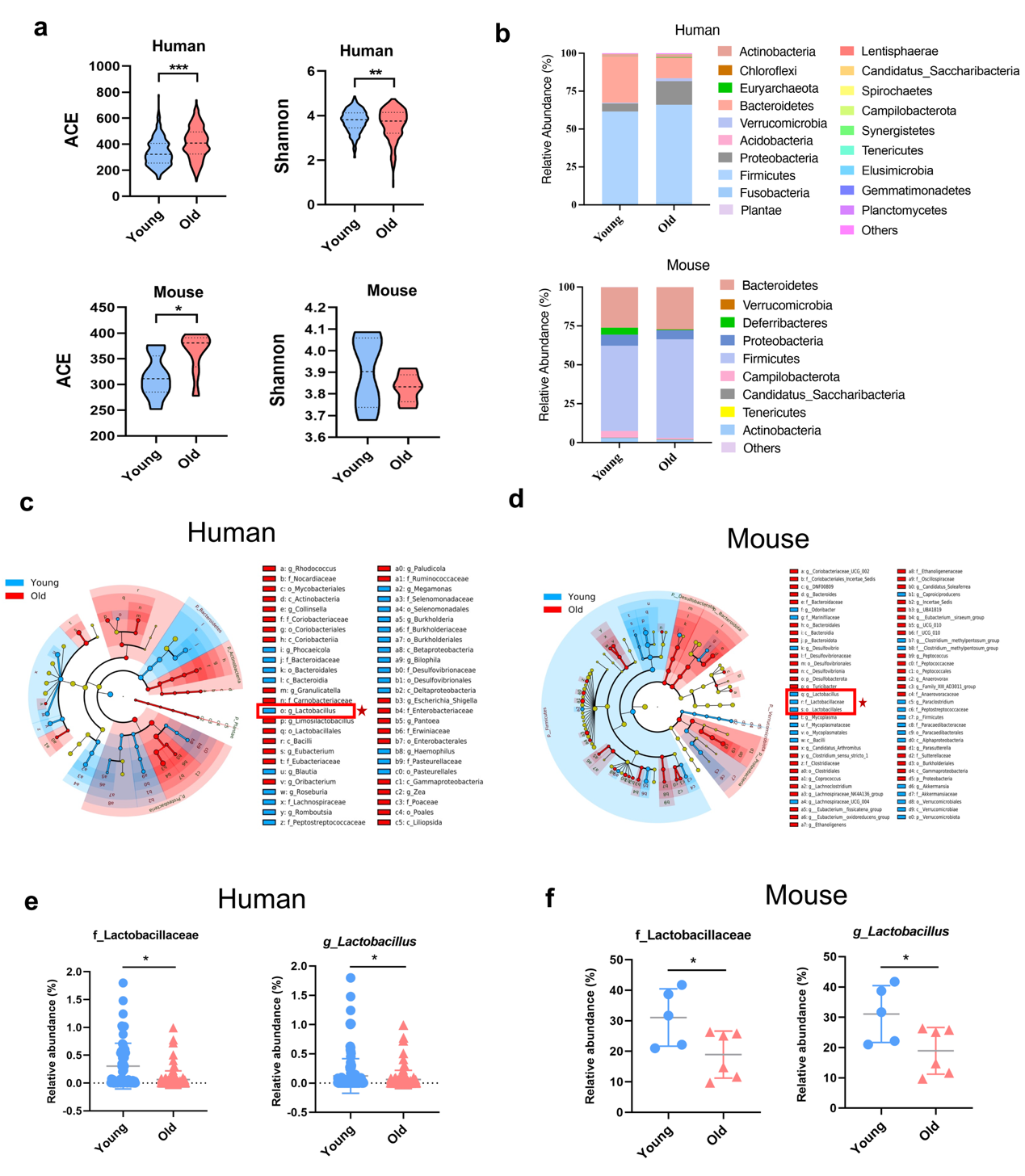
**

Figure S1. Gut microbial alterations during aging in human and mice. a) α-diversity analysis of fecal microbiota in young and old individuals, assessed by ACE and Shannon index. b) Relative abundance of gut microbial phylum in feces young (20–44 years, n = 314) and old (66–85 years, n = 386) human and cecal contents of young (2–3 months, n = 8) and old (19–20 months, n = 8) mice. c, d) LEfSe analysis showing differentially enriched taxa in old (red) vs. young (blue) individuals in humans (c) and mice (d). Red stars indicate the *Lactobacillaceae* family and *Lactobacillus* genus. e, f) Relative abundance of *f_Lactobacillaceae* and *g_Lactobacillus* in young and old human (e) and mouse (f) fecal samples. Data are shown as mean ± SD. *p < 0.05, **p < 0.01, ***p < 0.001 by Wilcoxon rank-sum test.

**
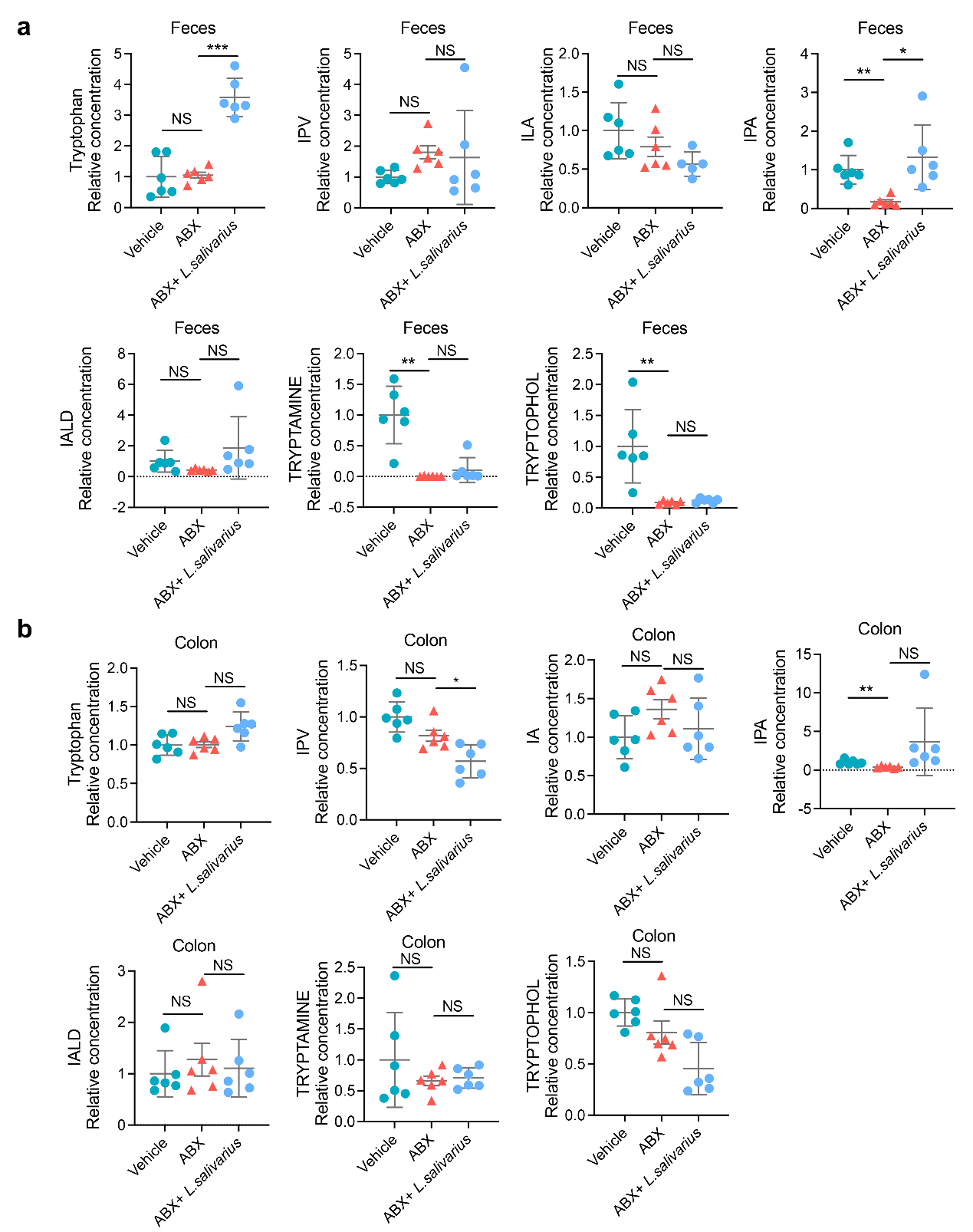
**

Figure S2. Effects of *L. salivarius* supplementation on fecal and colonic tryptophan metabolites in antibiotic-treated mice. a) Relative concentrations of tryptophan and its metabolites, including indole-3-propionic acid (IPA), indolelactic acid (ILA), Indole-3-acetaldehyde (IALD), tryptamine, and tryptophol, were measured in feces of mice treated with vehicle, antibiotics (ABX), or ABX followed by *L. salivarius* gavage. b) Relative concentrations of the same metabolites in colon tissue samples. Data are presented as mean ± SD (n = 6 per group). Statistical significance was assessed using one-way ANOVA followed by Tukey’s post hoc test. *p < 0.05, **p < 0.01, ***p < 0.001; NS, not significant.


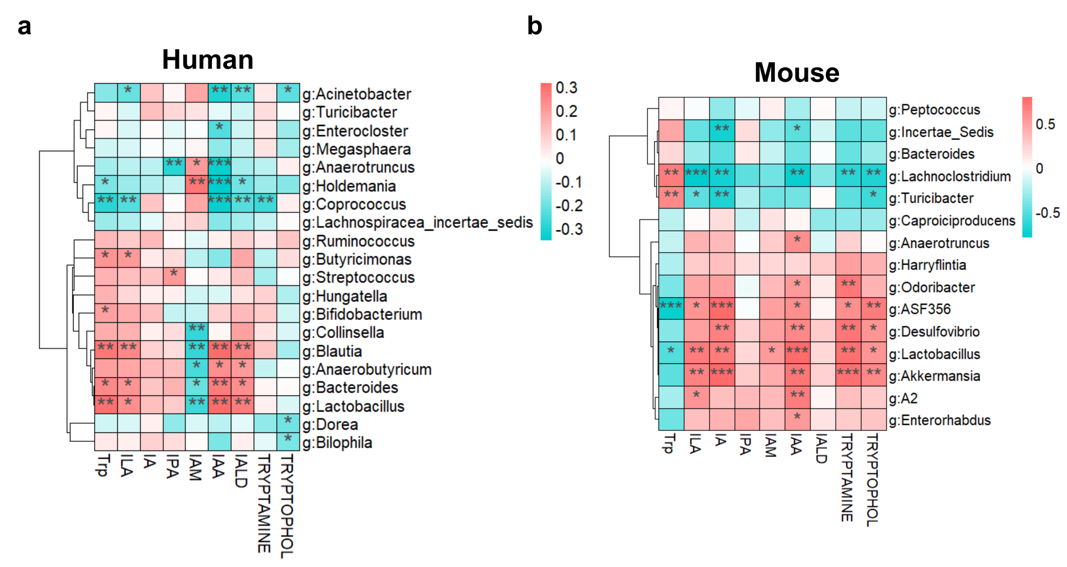


Figure S3. Correlation between gut microbial genera and fecal tryptophan metabolites in human and mice. a) Spearman correlation heatmap between the relative abundance of microbial genera and the levels of tryptophan (Trp) and its derivatives (ILA, IA, IPA, IAA, IAM, IALD, tryptamine, and tryptophol) in human fecal samples. b) Corresponding correlations in cecal contents of mouse. Color scale indicates the Spearman correlation coefficient with red and blue indicating positive and negative correlations, respectively. Asterisks denote statistical significance: * p < 0.05, ** p < 0.01, *** p < 0.001.


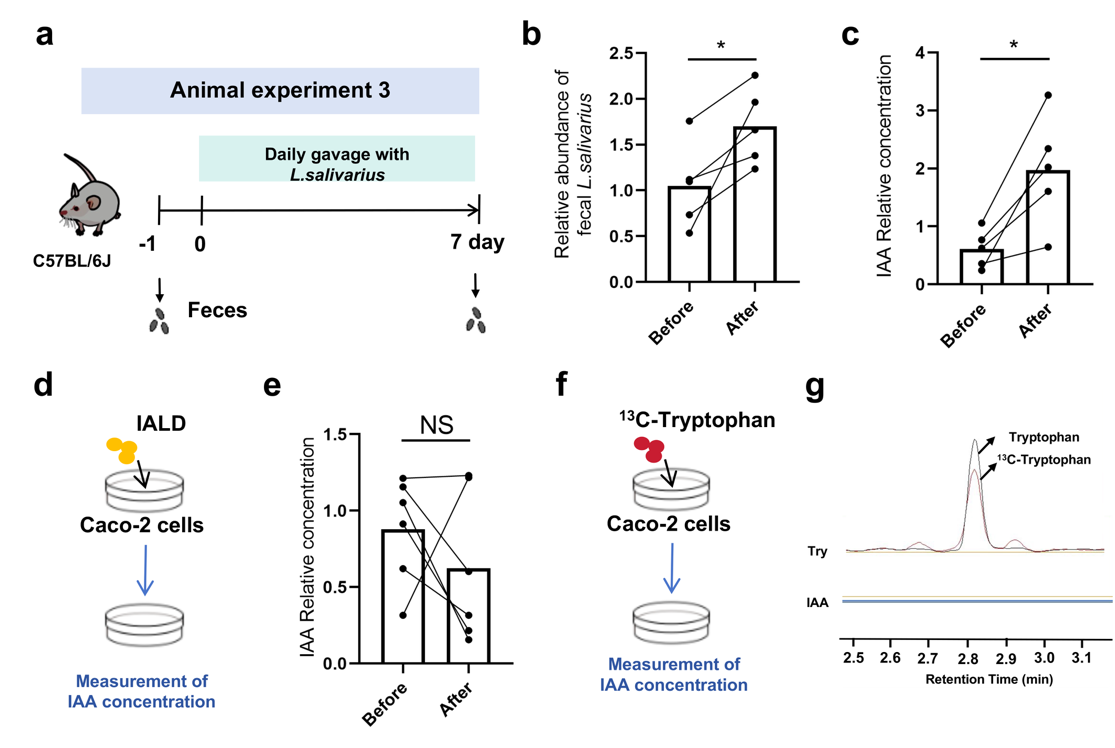


Figure S4. *L. salivarius* supplementation increases fecal IAA production in mice and host epithelial cells exhibit limited IAA synthesis capacity. a) Schematic diagram of the experimental design (n = 5): conventional mice were orally gavage with *L. salivarius* daily for 7 days to assess bacterial colonization and fecal IAA production. b) Relative abundance of fecal *L. salivarius* before and after 7 days of daily oral gavage in conventional mice. c) Relative fecal IAA concentrations before and after *L. salivarius* supplementation in conventional mice. d) Schematic diagram of Caco-2 cell treatment with indole-3-acetaldehyde (IALD) to assess IAA production capacity. e) Relative IAA concentrations in Caco-2 cell supernatants before and after IALD incubation (n = 6). f) Schematic diagram of Caco-2 cell treatment with isotopically labeled ^13^C-tryptophan. g) LC-MS measurement of tryptophan, ^13^C-tryptophan, and ^13^C-IAA in Caco-2 cells. Data are presented as mean ± SD. Statistical analysis was performed using paired Student’s t-test. *P < 0.05; NS, not significant.


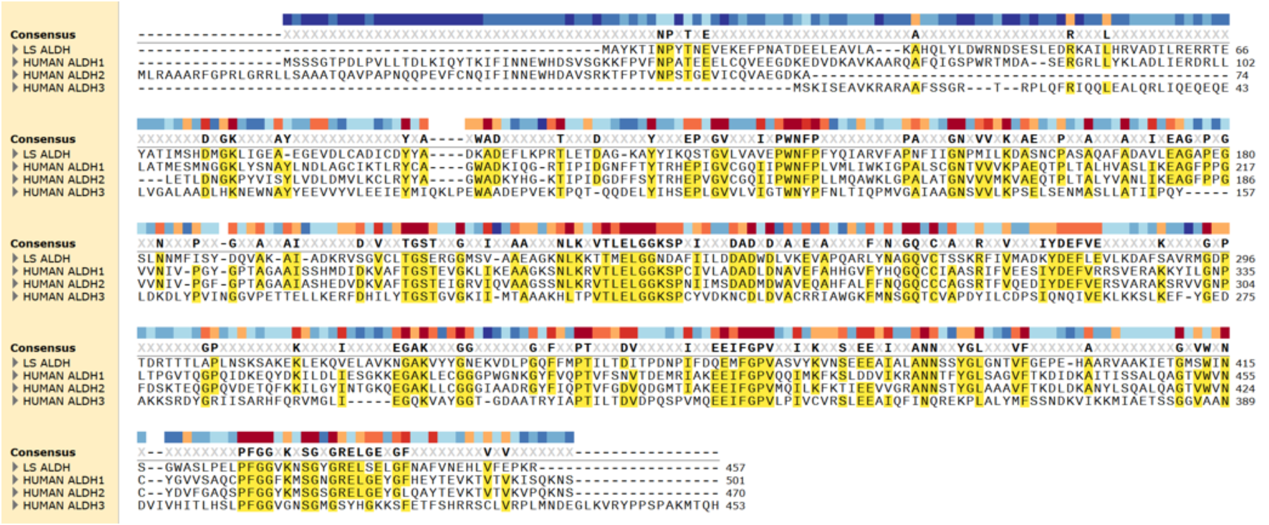
Figure S5. Multiple sequence alignment of *L. salivarius* ALDH and human ALDH isoforms. Amino acid sequence alignment of *L. salivarius* ALDH with human ALDH1, ALDH2, and ALDH3. Consensus sequences are indicated, with conserved residues highlighted. The alignment reveals partial sequence conservation and notable divergence across key domains, suggesting functional differences between bacterial and host ALDH enzymes.

**
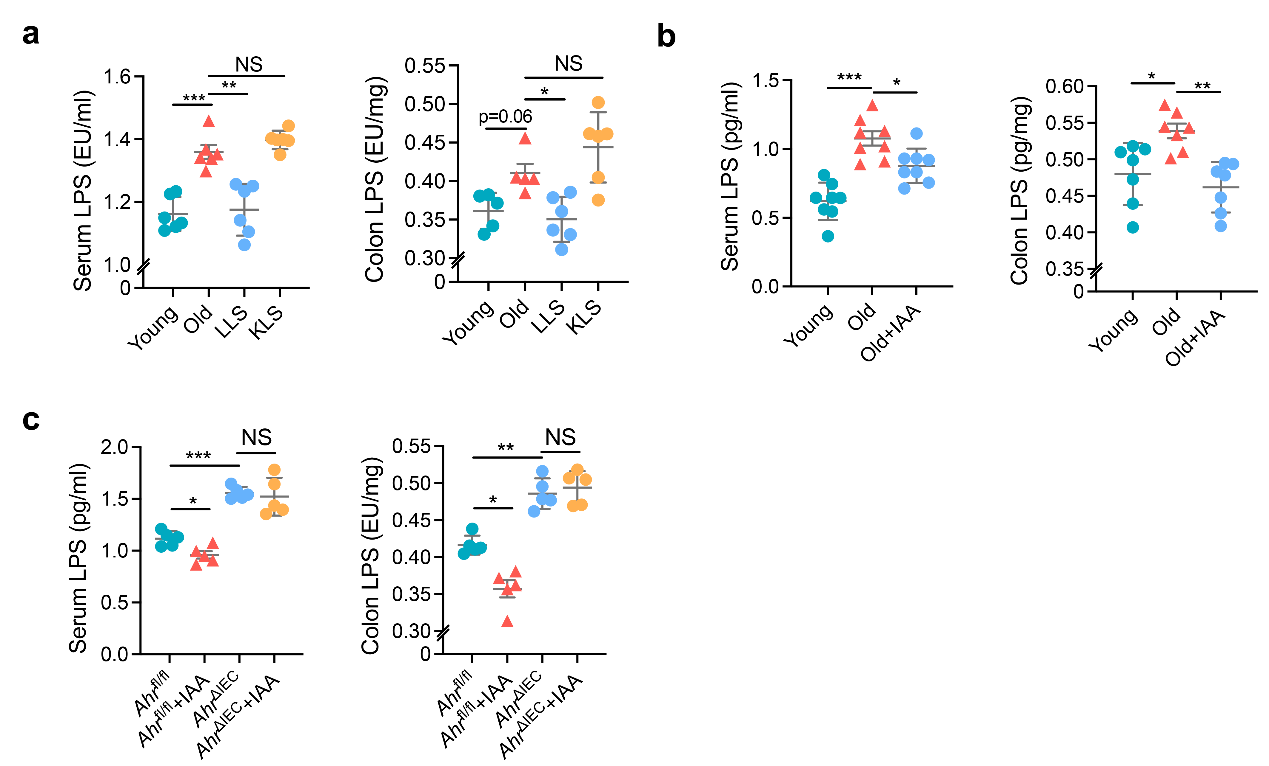
**Figure S6. *L. salivarius* and IAA reduce systemic LPS levels in aged mice. a) Serum and colonic lipopolysaccharide (LPS) levels were measured in young, old, and aged mice treated with live Lactobacillus salivarius (LLS) or heat-killed L. salivarius (KLS) for one month. LLS supplementation instead of KLS significantly reduced serum and colonic LPS concentrations compared to aged controls (n = 6). b) Serum LPS concentrations in young, old, and IAA-treated aged mice. IAA supplementation for two months significantly decreased systemic LPS levels in aged mice (n = 8). c) Serum and colonic LPS levels in aged *Ahr*^fl/fl^ and *Ahr*^ΔIEC^ mice with and without IAA supplementation (n = 5). The protective effect of IAA in lowering LPS was abolished in *Ahr*^ΔIEC^ mice, indicating that IAA-mediated barrier improvement requires epithelial AhR signaling. Data are presented as mean ± SD. Statistical significance was assessed using one-way ANOVA followed by Tukey’s multiple comparisons test. *P < 0.05, **P < 0.01, ***P < 0.001; NS, not significant.

**
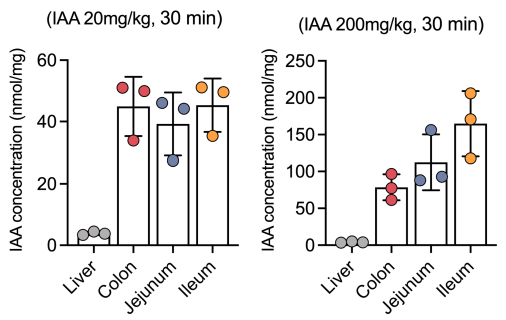
**

Figure S7. Pharmacokinetic profiling of IAA in intestine, colon and liver of mice upon oral exposure with IAA at different dosages.

**
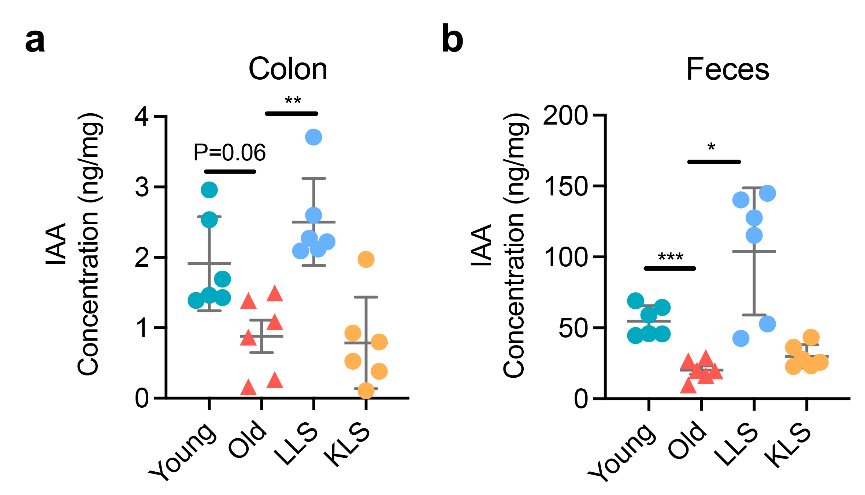
**

Figure S8. Live *L. salivarius* supplementation restores IAA levels in aged mice. a) Concentration of IAA in colon tissues from young, aged, and aged mice supplemented with live *L. salivarius* (LLS) and heat-killed *L. salivarius* (KLS). b) Concentration of IAA in fecal samples from the same groups. Data are presented as mean ± SD (n = 5). Each dot represents one biological sample. Statistical significance was assessed by one-way ANOVA with Tukey’s post hoc test. *P < 0.05, **P < 0.01, ***P < 0.001; NS, not significant.


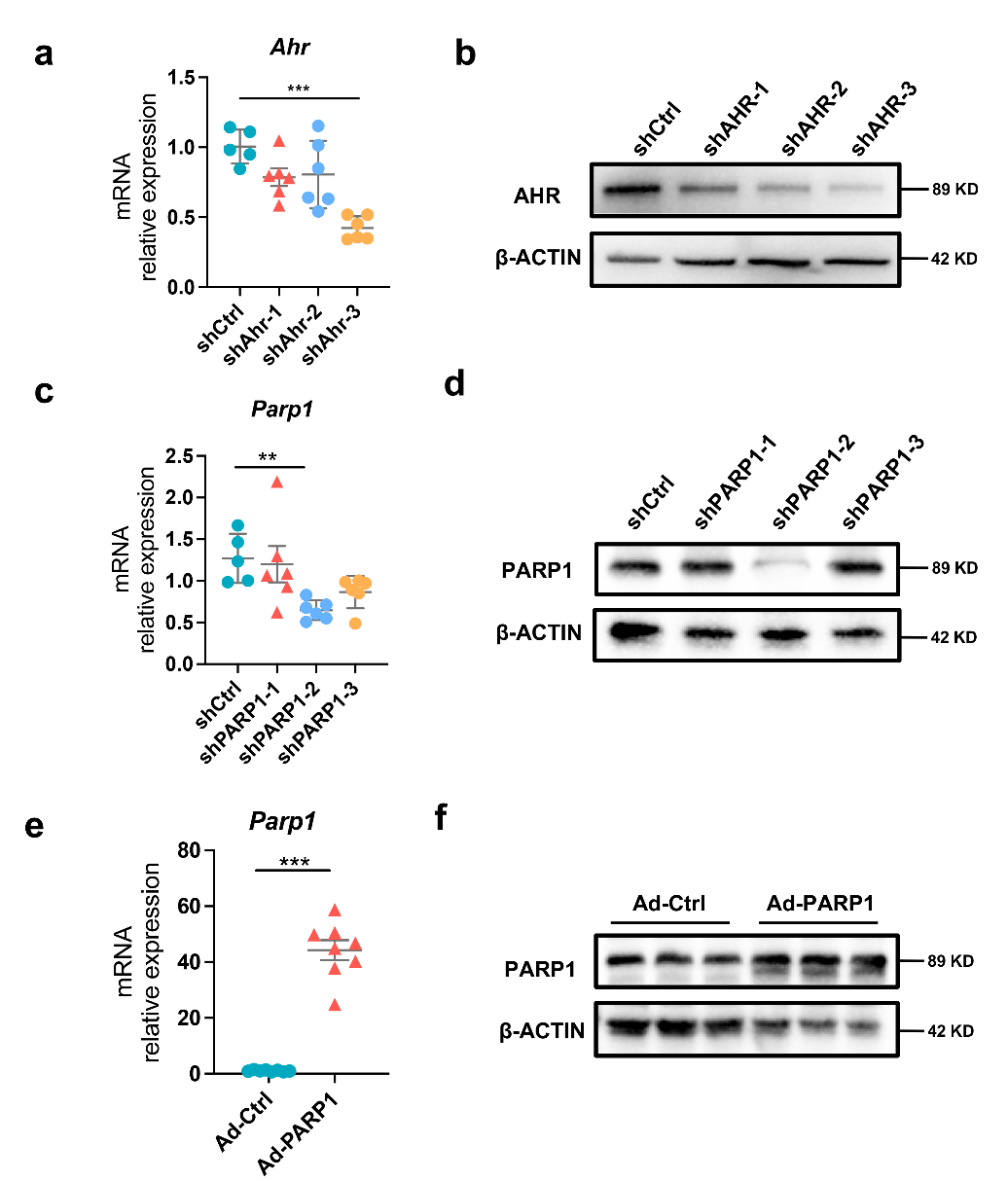


Figure S9. Validation of *Ahr* knockdown and *Parp1* knockdown/overexpression in Caco-2 cells. a,b) Quantification of mRNA and protein levels of AhR in cells transduced with non-targeting control (shCtrl) or three independent *Ahr*-targeting shRNAs (shAhr-1, -2, -3). c,d) Quantification of mRNA and protein levels of Parp1 following knockdown with three independent shRNAs (shParp1-1, -2, -3) compared to shCtrl. e, f) Quantification of mRNA and protein levels of Parp1 in cells infected with control adenovirus (Ad-Ctrl) or adenovirus expressing PARP1 (Ad-PARP1). β-ACTIN served as a loading control. Data are shown as mean ± SD (n = 3–6 per group). *p < 0.05, **p < 0.01, ***p < 0.001 by one-way ANOVA with Tukey’s post hoc test.

**
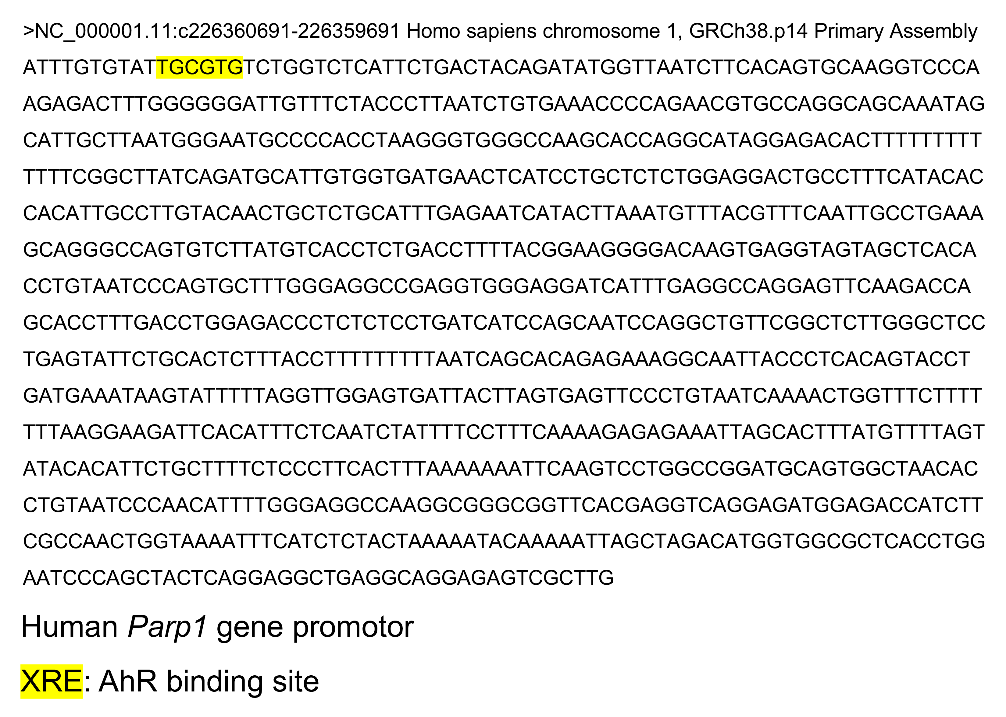
**

Figure S10. Identification of a putative AhR response element (XRE) in the mouse Parp1 gene promoter region. The DNA sequence shown corresponds to the promoter of *Parp1* gene in C57BL/6J mouse strain (NC_000001.11:c226360691-226359691). A canonical *Ahr* binding motif (XRE: 5'- TGCGTG-3') is highlighted in yellow, indicating a potential regulatory site through which *Ahr* may transcriptionally activate *Parp1* expression.

**Table S1. Sequence of primers**

| Transcript | Forward primer (5’-3’) | Reverse primer (5’-3’) |
| --- | --- | --- |
| *mParp1* | GGCAGCCTGATGTTGAGGT | GCGTACTCCGCTAAAAAGTCAC |
| *mP16* | AATCTCCGCGAGGAAAGC | GTCTGCAGCGGACTCCATS |
| *mP21* | ATTCCATAGGCGTGGGACCT | TCCTGGGCATTTCGGTCAC |
| *mMuc2* | AATGCCCTTGCGTCCATAAC | CAGGTGCAGGTATTGCAGTC |
| *mOccludin* | GGGCATTGCTCATCCTGAAG | GCCTGTAAGGAGGTGGACTT |
| *mE-cad* | CAGTTCCGAGGTCTACACCTT | TGAATCGGGAGTCTTCCGAAAA |
| *mClaudin* | GGCCCTGCCATCTTTATTGG | ATTCCCAGGACAGGAACAGG |
| *mZo-1* | TTCACGCAGTTACGAGCAAG | TTGGTGTTTGAAGGCAGAGC |
| *mPtprh* | GGTAAAAGTGGGTAGGAAATGGC | GTGGCTGTGTAGGACTGAGC |
| *mActin* | AGCCATGTACGTAGCCATCC | CTCTCAGCTGTGGTGGTGAA |
| *hParp1* | CGGAGTCTTCGGATAAGCTCT | TTTCCATCAAACATGGGCGAC |
| *hP16* | GGGTTTTCGTGGTTCACATCC | CTAGACGCTGGCTCCTCAGTA |
| *hP21* | TGTCCGTCAGAACCCATGC | AAAGTCGAAGTTCCATCGCTC |
| *hMuc2* | GAGGGCAGAACCCGAAACC | GGCGAAGTTGTAGTCGCAGAG |
| *hOccludin* | GCTACGGAAGTGGCTATGG | GCGGCAATGAAACAAAAG |
| *hE-cad* | GCCCATTTCCTAAAAACCTG | CTCTGTCACCTTCAGCCATC |
| *hClaudin* | CCTCCTGGGAGTGATAGCAAT | GGCAACTAAAATAGCCAGACCT |
| *hZo-1* | CAACATACAGTGACGCTTCACA | CACTATTGACGTTTCCCCACTC |
| *hPtprh* | GGCGGCACAACAGAGACTC | CTGTGGCAGTAGTGACAGTCC |
| *hActin* | CATGTACGTTGCTATCCAGGC | CTCCTTAATGTCACGCACGAT |

mouse primer (m), human primer（h）
